# Induction of cell death by sodium hexachloroplatinate (IV) in the HEI-OC1 cell line, primary rat spiral ganglion cells and rat organ of Corti explants

**DOI:** 10.1371/journal.pone.0307973

**Published:** 2024-07-26

**Authors:** Elisabeth Berger, Gudrun Brandes, Odett Kaiser, Janin Reifenrath, Thomas Lenarz, Kirsten Wissel, Martin Durisin

**Affiliations:** 1 Department of Otorhinolaryngology, Hannover Medical School, Hannover, Germany; 2 Lower Saxony Centre for Biomedical Engineering, Implant Research and Development (NIFE), Hannover Medical School, Hannover, Germany; 3 Hannover Medical School, Institute of Neuroanatomy and Cell Biology, Hannover, Germany; 4 Clinic for Orthopaedic Surgery, Hannover Medical School, Hannover, Germany; 5 University Clinic of Otolaryngology, Head and Neck Surgery, Otto-von-Guericke-University Magdeburg, Magdeburg, Germany; University of Louisville School of Medicine, UNITED STATES OF AMERICA

## Abstract

Although cochlear implants have become a well-established method for patients with sensory neural hearing loss, clinical results indicate that in some cases, corrosion of electrode contacts leads to high impedance that interferes with successful stimulation of the auditory nerve. As it is unclear whether corrosion products induce cell damage, we focused on cell culture models of the organ of Corti cell line (HEI-OC1), rat spiral ganglion cells (SGC) and rat organ of Corti explant (OC_ex_) cultivated from neonatal rat cochleae to characterize the cytotoxicity of sodium hexachloroplatinate (IV) (Na_2_(PtCl_6_)). The oxidative activity in HEI-OC1 cells decreased with increasing Na_2_(PtCl_6_) concentrations between 8 and 16 ng/μl, and live cell staining with Calcein acetoxymethyl/Ethidium homodimer III revealed an increasing number of cells with disrupted membranes. Ultrastructural evidence of mitophagy followed by necroptosis was detected. Additionally, exposure of the SGC to 15–35 ng/μl Na_2_(PtCl_6_) dose-dependently reduced neuronal survival and neuritogenesis, as determined by neurofilament antigen staining. In parallel, staining glial cells and fibroblasts with specific antibodies confirmed the dose-dependent induction of cell death by Na_2_(PtCl_6_). Exposure of the OC_ex_ to 25–45 ng/μl Na_2_(PtCl_6_) resulted in severe concentration-dependent hair cell loss. Our data show for the first time that Na_2_(PtCl_6_) induces cell death in a concentration-dependent manner in inner ear cell types and tissues.

## Introduction

Cochlear implantation remains the only therapeutic intervention for patients with profound sensory neural hearing loss to restore speech perception and enable verbal communication [1]. Insertion of the cochlear implant (CI) into the scala tympani causes trauma during electrode insertion, which leads to an increase in impedance due to possible mechanical damage to the lateral wall and basilar membrane, triggering inflammatory processes [2, 3]. However, clinical studies reported several cases of both spontaneous and recurrent impedance increases in the same channels that cannot be explained by inflammatory processes and technical failure alone [4, 5]. Scanning electron microscopy micrographs of cross sections of explanted electrodes revealed not only deposited connective tissue but also eroded platinum (Pt) surfaces [5–7]. Although Pt is the preferred electrode material for neuroprostheses due to its chemical inertness and biocompatibility, irreversible faradaic processes involving corrosion have been observed at electrode–electrolyte interfaces. These include both cathodic and anodic water hydrolysis, chloride ion oxidation, oxygen gas release and Pt oxidation [8–10]. It has been suggested that the most likely solute species at the Pt/saline interface are Pt^2+^ ions and their complexes with chloride oxidation products such as ClO^-^ and ClO^3-^ [8]. Also, time-of-flight secondary ion mass spectrometry (TOF-SIMS) analysis of the tissue-electrode interface of a patient’s CI revealed not only Pt oxide ions and their compounds with sodium and calcium but also the formation of Pt-protein complexes [6]. Additionally, Rosenberg et al. identified the octahedral [PtCl_6_]^2-^ complex generated during the electrolysis of Pt electrodes as a consequence of acidification of the chloride solution [11].

In addition to ionic Pt compounds, traces of particulate Pt have been detected in animals after several weeks of electrical stimulation [12, 13] and in long-term CI users, mainly in electrode tissue capsules [6, 14, 15]. One reason for the formation of solid particles could be the oxidation of Pt to [PtCl_6_]^-2^ in the presence of chloride ions during the anodic pulse and the reduction to elemental Pt under cathodic conditions [8, 16].

Numerous studies have reported that Pt compounds induce inflammation, oxidative and endoplasmic reticulum (ER) stress and DNA damage. For example, cell division in *Escherichia coli* bacteria was suppressed following Pt electrode corrosion [11]. Furthermore, it was discovered that cis-diamminetetrachloroplatinum (IV), cis-[PtCl_4_(NH_3_)_2_], appeared as the most effective inhibitor of bacterial cell division [17]. Other studies have mainly investigated the ototoxicity of Pt-based chemotherapeutics in animals, auditory cells and tissues, and tumor cell lines and tissues [18, 19]. A recent study demonstrated *in vitro* cell death induction in NIH 3T3 and SH-SY5Y cells lines the administration of ionic Pt generated by the electrochemical stimulation of a human cochlear electrode [20]. However, the interactions of Pt compounds other than those derived from antineoplastic Pt compounds with inner ear-related cells and tissues have not yet been examined. Since cell damage induction by corrosion products has still not been proven, this study aimed to characterize the cytotoxicity of sodium hexachloroplatinate (IV) (Na_2_(PtCl_6_)) on cell culture models of the mouse organ of the Corti cell line (HEI-OC1), spiral ganglion cells (SGC) and organ of Corti explants (OC_ex_) cultivation, both of which were dissected from neonatal rat cochleae. Na_2_(PtCl_6_) was used as a water-soluble and chemically stable model Pt(IV) complex, representing one of the Pt(IV) compounds potentially generated during Pt corrosion *in vivo*. The authors hypothesized that Na_2_(PtCl_6_) may be involved in the induction of cell death in inner ear-specific cells in a similar manner to that demonstrated in the NIH 3T3 and SH-SY5Y cell lines [20].

## Methods

### Dispersion of Na_2_(PtCl_6_)

A 500 mg/ml stock solution of Na_2_(PtCl_6_) (Sigma‒Aldrich, Taufkirchen, Germany) was prepared in sterile double distilled water. Na_2_(PtCl_6_) working solutions were obtained after dilution in high-glucose Dulbecco’s Modified Eagle’s Medium (DMEM, Bio&Sell, Germany) supplemented with 10% fetal calf serum (FCS, Bio&Sell, Germany) at concentrations between 8 and 45 ng/μl. Based on previous studies using cisplatin and ionic Pt obtained by electrical stimulation of a human CI to induce cell death in HEI-OC1 [21] and NIH 3T3 as well as SH-SY5Y cell lines [20], the concentration range for each experimental approach was determined by the range in which an effect was observed.

### Seeding and culturing of HEI-OC1 cells following Na_2_(PtCl_6_) supplementation

The HEI-OC1 cell line was kindly provided by Michael Morgan and Axel Schambach (Institute of Experimental Hematology, Hannover Medical School, Hannover, Germany). As described previously [22], 4000 cells/100 μl of DMEM supplemented with 10% FCS were precultivated for 24 h at 33°C in 10% CO_2_, followed by replacement of the cell culture medium containing Na_2_(PtCl_6_) at concentrations of 8, 10, 12, 14 and 16 ng/μl and cultivation for an additional 48 h. Statistical evaluation was performed on N = 10 experiments, and each assay was performed in triplicate (n = 3). HEI-OC1 cell culture assays without Na_2_(PtCl_6_) incubation were used as a reference for relative quantification of samples exposed to Na_2_(PtCl_6_). Cell culture assays in which 15% DMSO was administered for 1.5 h served as a cell death control. Cellular changes in HEI-OC1 cells were documented using a transmission light microscope (Olympus CKX41SF, Hamburg, Germany) equipped with a CCD color camera (Olympus Color View III, Hamburg, Germany).

### Resazurin assay for examination of the oxidative activity of HEI-OC1 cells after exposure to Na_2_(PtCl_6_)

A VisionBlueTM Quick Cell Viability Fluorometric Assay Kit (BioVision, Mountain View, CA, USA) was used for relative quantification of the metabolic activity of the HEI-OC1 cells exposed to varying concentrations of Na_2_(PtCl_6_). In untreated cell culture assays, water-soluble, non-fluorescent resazurin is reduced by dehydrogenases to resorufin. The resulting fluorescent signal intensities are directly proportional to the oxidative activity of the cells. Toxic compounds interfere with the reduction of resazurin, resulting in a decrease in signal intensity. The cell culture medium was replaced with medium containing 10% resazurin solution, and the cells were incubated for 2.5 h at 33°C. Fluorescence was measured at 550/600 nm excitation/emission wavelengths using a microplate reader (Synergy H1, Biotek, Bad Friedrichshall, Germany). Cell culture medium prepared with resazurin alone was used as a background control. For data analysis, the signal intensities of Na_2_(PtCl_6_)-treated samples were related to untreated cells as a reference and calculated as percentages (%). A cell viability of less than 70% relative to the reference was defined as cytotoxic according to ISO 10993–5:2009, as previously described [22].

### Ethidium homodimer III (EthD) and Calcein acetoxymethyl (Calcein AM) labeling of HEI-OC1 cells exposed to Na_2_(PtCl_6_)

To distinguish between live and dead cells in the same cell population after Na_2_(PtCl_6_) addition, a viability/cytotoxicity assay with Calcein AM (green) and EthD (red) dyes (Biotium, Fremont, CA, USA) was used with slight modifications [22]. Unlike the membrane-permeable dye Calcein AM, which is a DNA-intercalating dye, EthD can only enter cells with disrupted membranes upon the induction of cell death. Vital cells trigger the cleavage of Calcein AM in the cytoplasm by activating esterases, followed by the formation of green fluorescent Ca^2+^-calcein complexes that are unable to escape from the cytoplasm. According to the protocol of Berger et al., 2 μM Calcein AM and 4 μM EthD were added to the serum-free cell culture medium, and 30 μl of the staining solution was added to each sample [22]. After incubation for 30–45 min at room temperature, the samples were evaluated by using a fluorescence microscope (Keyence BZ 9000 Biorevo, Keyence International, Mechelen, Belgium).

### Transmission electron microscopy

To investigate the effects of Na_2_(PtCl_6_) on cellular ultrastructures, HEI-OC1 cells were seeded at a density of 300,000 cells per well in a 6-well microtiter plate (Nunclon, Thermo Fisher Scientific, Kempen, Germany) and cultured in 3 ml of high-glucose DMEM for 24 h as described above. Cultivation proceeded for 48 h either in 3 ml of culture medium alone or spiked with 8–16 ng/μl Na_2_(PtCl_6_). After incubation, the medium was removed and replaced with phosphate buffered saline (PBS). As previously described [22], the cells were detached with a cell scraper and centrifuged at 1000 rpm for 4 min (MiniSpin Plus, Eppendorf, Hamburg, Germany). The supernatant was discarded and the cell pellet was fixed with 2.5% glutardialdehyde (Polysciences, Warrington, PA, USA) in 0.1 M sodium cacodylate (Th. Geyer, Hamburg, Germany). The samples were postfixed in 2% osmium tetroxide (Polysciences) in 0.1 M sodium cacodylate, dehydrated in graded ethanol (Baker, Phillipsburg, NJ, USA), and embedded in epoxy resin (Serva, Heidelberg, Germany). Ultrathin sections were prepared and stained with 2% uranyl acetate (Serva) and lead citrate (Serva) and examined with a transmission electron microscope (Morgagni 268, 80 kV, Eindhoven, The Netherlands). The digital images were processed with Adobe Photoshop CS6 (Adobe Systems Software Ireland Limited). Concentration ranges were investigated in which cell pellets predominantly contained living cells.

### Ethical statement

Neonatal Sprague-Dawley rats (postnatal day 3–5, n = 10–12 animals) were used for each preparation of the spiral ganglia (SG) and the OC_ex_ preparation. All experiments and analyses in this study were performed in accordance with the institutional guidelines for animal welfare of the Hannover Medical School. These guidelines comply with the standards of the German Animal Welfare Act and with the European Directive 2010/63/EU on the protection of animals used for experimental purposes. The use of animals exclusively for tissue studies is registered with the competent authority (Central Animal Laboratory of the Hannover Medical School, including an institutional animal care and use committee) in accordance with legal requirements (No. 2018/215) and reported regularly as required by law. As no other treatment is performed before the animals are killed, no further authorization is required (§4 Animal Protection Act).

### Dissection and dissociation of spiral ganglia containing neurons, fibroblasts and glial cells

SG dissection and SGC dissociation were performed as previously described [22]. The rats were decapitated, the skin was detached from the skull, and the mandibula was removed. The skull was divided into two halves, and both halves were immersed in ice-cold PBS (Invitrogen, Thermo Fisher, Karlsruhe, Germany) to dissect the spiral ganglion under a microscope (Leica MZ-6, Bensheim, Germany). After opening the bony cochlear capsule, the organ of Corti (OC) and the stria vascularis were removed from the modiolus, and the spiral ganglia were collected in ice-cold Ca^2+^/Mg^2+^-free Hank’s balanced salt solution (HBSS, Invitrogen).

Enzymatic dissociation was conducted in 2 ml of prewarmed Ca^2+^/Mg^2+^-free HBSS (Invitrogen) supplemented with 0.1% trypsin (Serva, Heidelberg, Germany) and 0.01% DNase I (Roche, Mannheim, Germany) for 10–12 min at 37°C [23]. Following the addition of 200 μl of FCS (Bio&Sell, Feucht, Germany) to interrupt enzymatic activity, cell clusters were washed three times in serum-free neuromedium (stock solution: Panserin 401, PAN Biotech GmbH, Aidenbach, Germany; 1 M 4-(2-hydroxyethyl)-1-piperazineethanesulfonic acid, HEPES, Gibco, Thermo Fisher Scientific, Waltham, USA; 10 mg/ml PBS, Invitrogen; 30 iE/ml penicillin, Sigma-Aldrich; 40% glucose solution diluted to 30% in PBS, Braun AG, Melsungen, Germany; 4 mg/ml insulin, Sigma-Aldrich; 0.1 μg/ml N2 supplement, Gibco) and mechanically dissociated. The final cell number was determined by counting the SGC in the Neubauer cell counting chamber following staining of the cells with 10% trypan blue (Sigma-Aldrich).

### Dissection of the OC and organotypic cultivation

For organotypic cultivation, dissection of the inner ears was performed as described above. After the OC (denoted as OC_ex_) was peeled off from the modiolus, the OC_ex_ was transferred within a droplet of medium onto floating membranes (SPI-Pore Polycarbonate Track Etch Membrane Filters, SPI supplies, West Chester, USA) using a pipette with a cut tip, followed by alignment and uncoiling under a microscope [24]. The membranes were preincubated in 500 μl completed DMEM/F12 (buffered with HEPES, PAN Biotech), MACS^®^ NeuroBrew-21^®^ (Miltenyi Biotec, Bergisch Gladbach, Germany), 1 mM NAC (N-acetyl-L-cysteine, Sigma-Aldrich, Merck, Darmstadt, Germany), 5 ng/ml EGF (human recombinant epidermal growth factor; Gibco), 2.5 ng/ml bFGF (basic fibroblast growth factor, Gibco) and 67 mg/ml penicillin (Sigma-Aldrich) for approximately 30 min in 4-well cell culture plates (Nunclon). Following removal of the medium, the OC_ex_ were supplemented with 25–45 ng/μl Na_2_(PtCl_6_) in completed DMEM/F12 and cultivated for an additional 72 h at 37°C and 5% CO_2_. OC_ex_ cultivation in medium alone was used as an experimental control. The explants were washed with PBS prior to fixation with 500 μl of 4% PFA in PBS (paraformaldehyde granulated extra pure, Carl Roth, Karlsruhe, Germany) for 30 min at room temperature.

### Seeding and cultivation of SGC exposed to varying Na_2_(PtCl_6_) concentrations

As previously described [22, 25], 96-well microtiter plates were coated with 0.1 mg/ml Poly-DL-Ornithine (Sigma-Aldrich) and 0.01 mg/ml laminin (Invitrogen). Following SGC seeding (1 x 10^4^ cells/50 μl of serum-free neuromedium), Na_2_(PtCl_6_) was administered to the cell culture assays at concentrations ranging from 15 ng/μl to 35 ng/μl in a neuromedium containing 10% FCS at the final concentration. SGC cultivation without Na_2_(PtCl_6_) supplementation was used for relative quantification of neuronal survival and neurite length. An SGC assay containing 2.5% DMSO was included as a cell death control for this cell culture model. Data analysis was performed using N = 5 independent experiments, with each assay performed in triplicate. The SGC were cultivated at 37°C and 5% CO_2_ for 48 h, followed by fixation with 1:1 methanol/acetone.

### Immunofluorescence for examination of the spiral ganglion neuron (SGN) survival rate, neurite outgrowth and SGC composition

Indirect immunofluorescence detection of cell-specific antigens was used for relative quantification of the survival rate, examining the neurite outgrowth of the SGN and the cellular composition of the SGC following exposure to varying Na_2_(PtCl_6_) concentrations, as recently described [22]. Table 1 lists the primary and secondary antibodies used in this study. According to the descriptions in the table, primary and secondary antibodies were diluted in PBS containing 1% bovine serum albumin (BSA, Serva, Heidelberg, Germany). Positively labeled antigens were detected and analyzed by fluorescence microscopy using Keyence BZ 9000 Biorevo (Keyence International, Mechelen, Belgium) and Zeiss Axio Observer Z1 (Zeiss, Jena, Germany). For quantitative determination of the survival rate and the neurite lengths of the neurons, images were digitally captured (Axiocam MRm, Zeiss, Jena, Germany) and analyzed by using PALM RoboSoftware (Palm Zeiss, Munich, Germany). Survival rates were calculated as the number of SGN exposed to Na_2_(PtCl_6_) compared to the number of SGN not treated with Na_2_(PtCl_6_) (in %). Only SGN with neurites of at least three times the average cell diameter were included for statistical evaluation, and the 5 longest neurites of each sample were considered for data collection.

**Table 1:** Primary and secondary antibodies for immunostaining of cell-specific antigens in the SGC.

**Primary antibody**	**Host**	**Description**	**Specificity**	**Manufacturer**	**Dilution**
Neurofilament 200 kD, monoclonal	Mouse	Intermediary filament	Neurons	Novocastra	1:400
				#NCL-NF200	
p75, polyclonal	Rabbit	Neurotrophic growth	Glial cells	Abcam	1:500
				#38335	
Vimentin clone V9,	Mouse	Intermediary filament	Fibroblasts	Dako	1:200
monoclonal			Glia cells	#M0725	
**Secondary antibody**	**Host**	**Description**	**abs/em [nm]**	**Manufacturer**	**Dilution**
**IgG (H+L)**					
Anti-mouse	Goat	New Dylight 488	493/518	Jackson-	1:400
		(GaM 488)		Immunoresearch	
				#115-485-008	
Anti-rabbit	Goat	Alexa Fluor 594	591/616	Jackson-	1:100
				Immunoresearch	
				#111-515-144	

### Phalloidin staining of the OC_ex_

Staining with fluorescence-labeled phalloidin was performed to detect the loss of hair cells in the OC_ex_ in response to Na_2_(PtCl_6_) exposure. The fixed explants on the membranes were washed with PBS for 2 min, permeabilized with 0.2% Triton X-100 in PBS for 10 min, washed again with 0.1% Triton X-100 in PBS for 2 min and blocked with 5% FCS in PBS for 1 h.

Subsequently, the explants were stained with Alexa Fluor^TM^ 488 Phalloidin (Invitrogen) diluted 1:100 in 0.5% BSA and 0.1% Triton-X in PBS for 45 min at room temperature. The samples were washed with PBS once for 1 min and twice for 5 min each. As follows, the OC_ex_ were carefully loosened from the membranes using a forceps leg and transferred into lens-sized drops of mounting medium (ProLongGold, Invitrogen), which were subsequently placed onto coverslips (Epredia, Microm International GmbH, Dreieich, Germany). Prior to final embedding with another coverslip, the explants were aligned under a microscope (Leica MZ-6, Bensheim, Germany). After curing, the OC_ex_ were analyzed using a laser scanning confocal microscope (Leica Microsystems GmbH, Wetzlar, Germany) equipped with a white light laser combined with Leica confocal software (LAS X Science Microscope Software; version LAS X 3.5.7.23225). Image acquisition was performed at λ = 488 nm and 100x magnification using HCX PL APO 100x/1.40 OIL (Leica) prepared with immersion oil type F (Leica). The images were acquired using z-stacks (average of 25 individual exposures) and processed using ImageJ software (Wayne Rasband, National Institutes of Health, Bethesda, MD, USA).

### Statistical analysis

All data from *in vitro* and *ex vivo* cell culture assays are presented as mean ± standard error of the mean (SEM). Nonparametric repeated measures analysis of variance (repeated measures ANOVA) and Tukey’s multiple comparison test were used for statistical analysis. In all the statistical analyses, P < 0.05 was set as the threshold for significance.

## Results

### Na_2_(PtCl_6_) induces concentration-dependent cell death in the HEI-OC1 cell line

**Fig 1:**
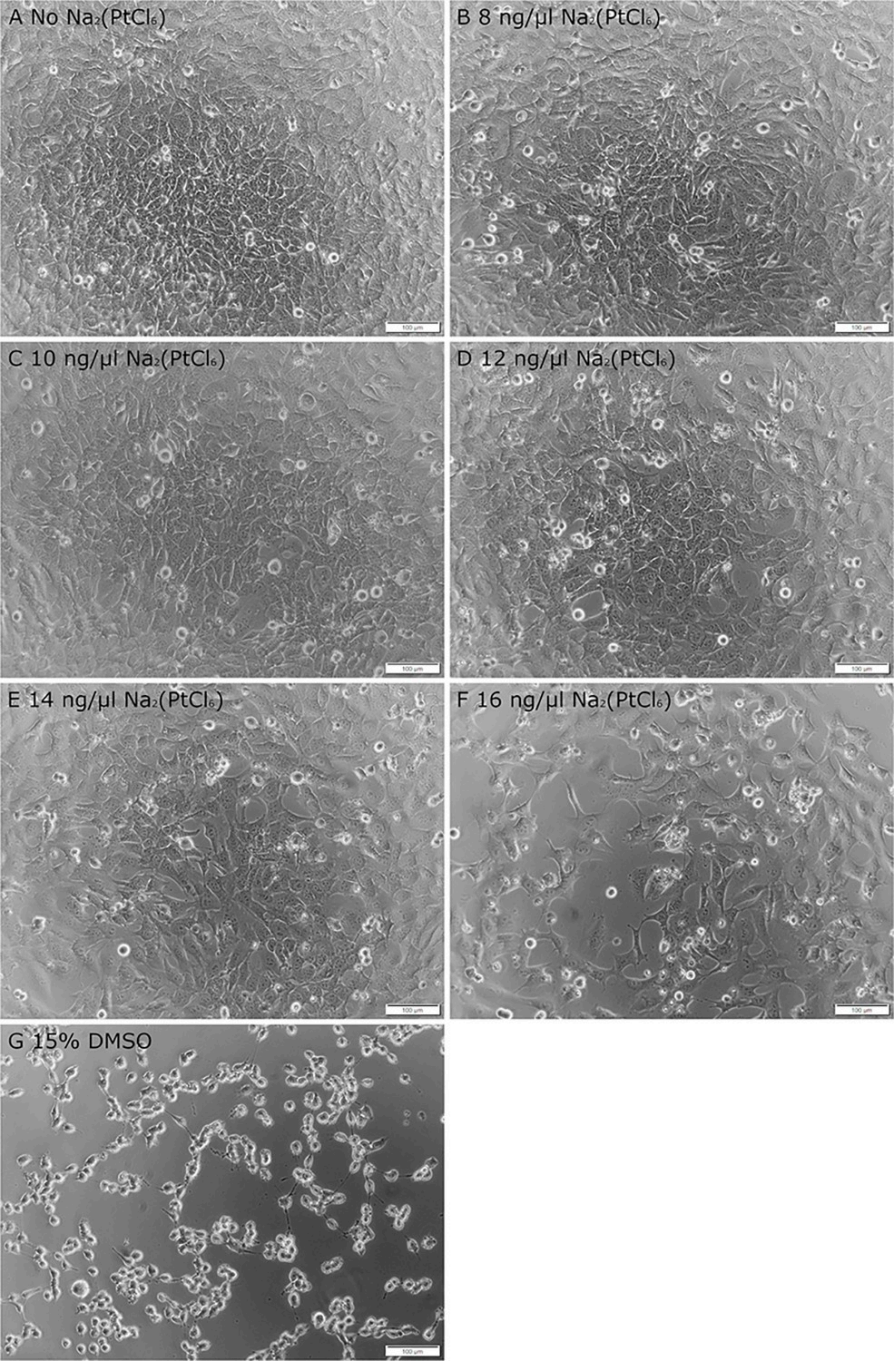
Representative microscopic view of the morphology of the HEI-OC1 cells following exposure to Na_2_(PtCl_6_). HEI-OC1 cells were cultivated either without Na_2_(PtCl_6_) as an experimental control (A) or in culture media supplemented with 8 ng/μl (B), 10 ng/μl (C), 12 ng/μl (D), 14 ng/μl (E) or 16 ng/μl of Na_2_(PtCl_6_) (F). HEI-OC1 cells incubated with 15% DMSO for 1.5 h served as a cell death control (G). Normal cell growth without any morphological anomalies could be observed in the experimental control, whereas cell culture assays exposed to Na_2_(PtCl_6_) resulted in a concentration-dependent decrease in cell density and adhesion as well as loss of cell contacts. Microscopic images shown are representative of a total of N = 10 experiments. Each experiment was performed in triplicate (n = 3). Scale bars: 100 μm.

First, the morphology of the HEI-OC1 cells following exposure to Na_2_(PtCl_6_) at concentrations between 8 ng/μl and 16 ng/μl was examined microscopically. Non-treated cells exhibited well-developed dendrites, flat spreading lamellipodia, a round nucleus and physiological cell growth (Fig 1A), whereas samples exposed to Na_2_(PtCl_6_) showed signs of cellular disintegration from 8 ng/μl: Administration of 8–12 ng/μl Na_2_(PtCl_6_) led to moderate morphological changes, vacuole formation and cell loss (Fig 1B–1D), while Na_2_(PtCl_6_) concentrations greater than 12 ng/μl resulted in severe loosening of the intercellular contacts as well as their focal adhesion points in a dose-dependent manner (Fig 1E and 1F). As a consequence, normally growing flat cells with broad lamellipodia became floating spherical cells partially surrounded by blebs. As shown in Fig 1G, cell culture assays using 15% DMSO served as a cell death control showed predominantly spherical HEI-OC1 cells.

**Fig 2:**
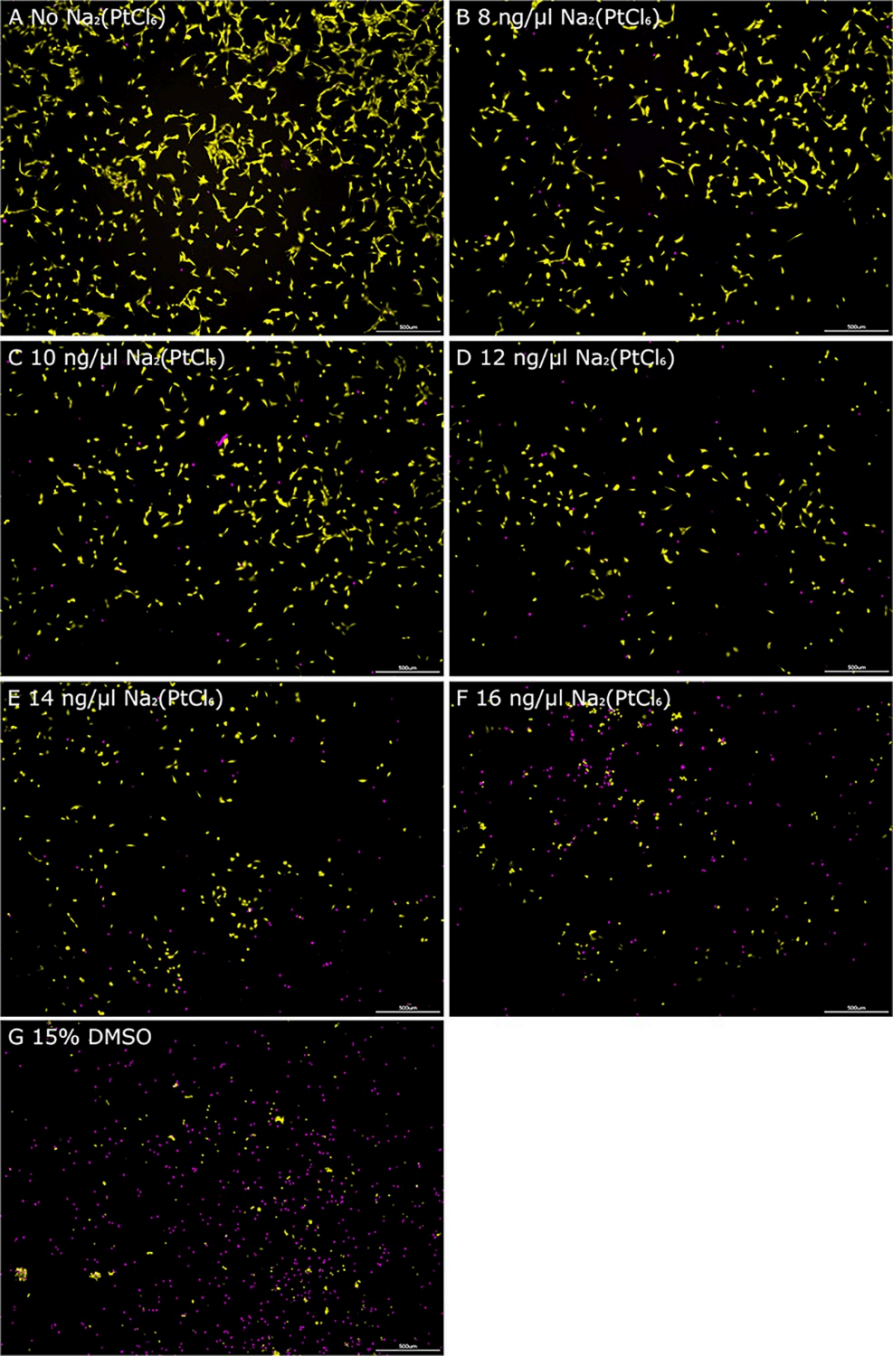
Representative live cell staining of HEI-OC1 cells for evaluation of cell death in presence of Na_2_(PtCl_6_). Calcein AM (green fluorescence, stained in false colour yellow in the images) and EthD (red fluorescence, stained in false colour magenta in the images) were used for fluorescent live cell staining following Na_2_(PtCl_6_) exposure. The plasma membrane-permeable Calcein AM represents vital following its cleavage to calcein in the cytoplasm and conversion to the green fluorescent dye in the presence of Ca^2+^ ions. In contrast, EthD is only able to infiltrate cells with leaky membranes to intercalate double-stranded DNA emitting red fluorescent light. HEI-OC1 cells cultivated without Na_2_(PtCl_6_) represented the experimental control group showing any cell loss (A), whereas those exposed to 8 ng/μl (B), 10 ng/μl (C), 12 ng/μl (D), 14 ng/μl (E) and 16 ng/μl (F) Na_2_(PtCl_6_) demonstrated dose-dependent cell death induction, as indicated by an increase in red fluorescent dots and a decrease in vital cells monitored by Calcein AM triggered green fluorescence. Similarly, DMSO-induced cytotoxicity in the HEI-OC1 cells could be shown by red fluorescent EthD-DNA complexes as a result of membrane disintegration (G). The images of live cell staining of the HEI-OC1 cells are representative of a total of N = 3 experiments. Each experiment was performed in triplicate (n = 3). Scale bars: 200 μm.

Dose-dependent cell toxicity was verified by staining vital cells with Calcein AM and EthD (Fig 2). The untreated cell group presented mainly vital cells with well-defined filopodia adhering to the surface of the cell culture dish, as demonstrated by Calcein AM staining, which retained in the cytosol after cleavage to calcein by esterases (Fig 2A). Only a few cells were penetrated by EthD, which intercalated the DNA strands due to disruption of the plasma membrane. After the application of Na_2_(PtCl_6_), a concentration-dependent increase in the number of partially detached dots representing dying cells with ruptured membranes was observed (Fig 2B–2F). In addition, exposure of HEI-OC1 cells to Na_2_(PtCl_6_) from 8 ng/μl up induced stress-induced morphological changes, as described above (Fig 2B). The higher the concentration of the Pt compound, the more cells retracted their cell contacts as well as focal adhesion points, forming spherical structures. Similar to the transmission light analysis of the DMSO-treated group, the majority of the HEI-OC1 cells underwent cell death (Fig 2G).

**Fig 3:**
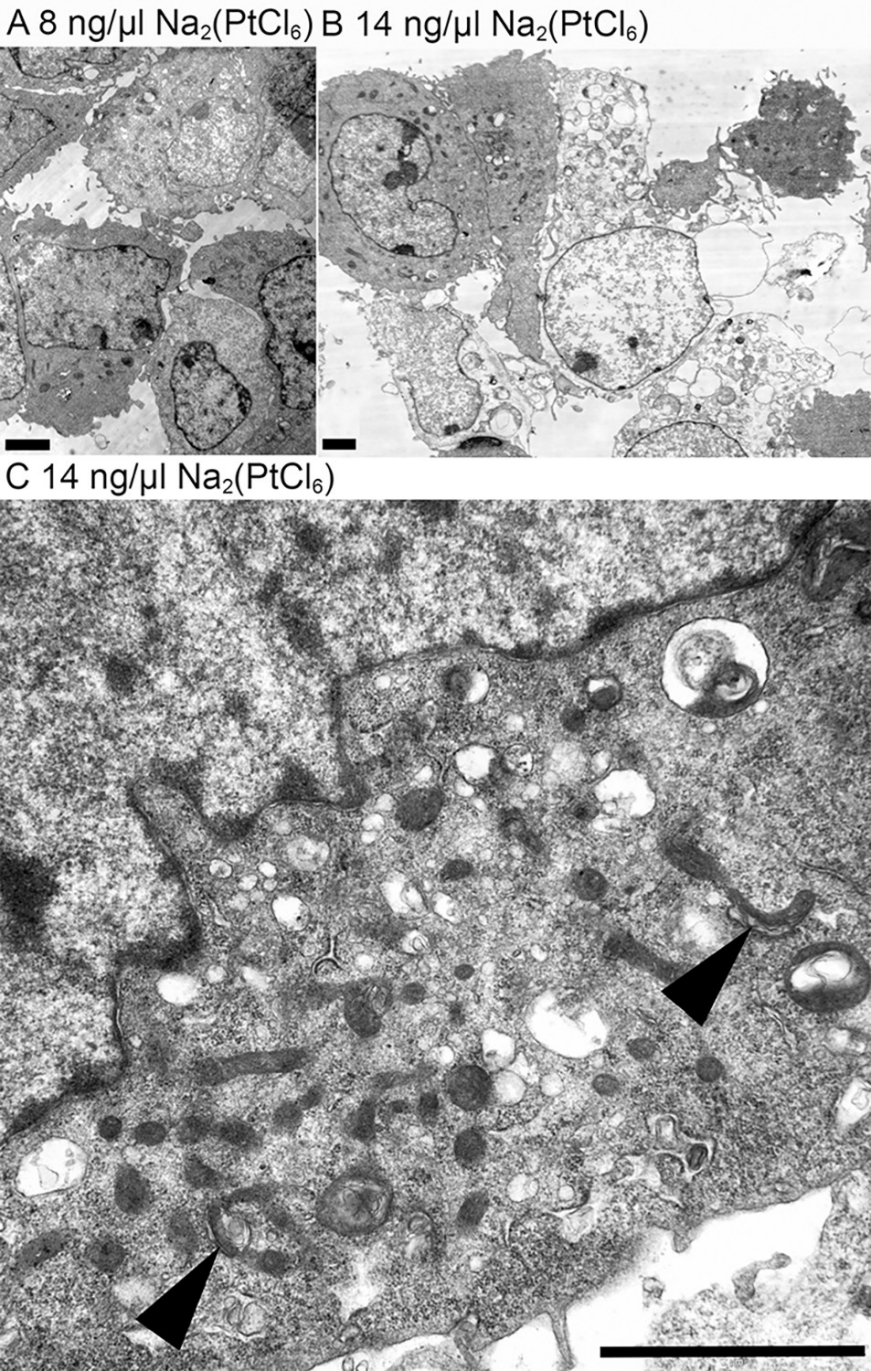
Presence of mitophagy in HEI-OC1 cells following exposure to Na_2_(PtCl_6_). The normal ultrastructure of the HEI-OC1 cells also remained unchanged in the presence of lower doses of Na_2_(PtCl_6_) (8 ng/μl Na_2_(PtCl_6_)) (A). Following incubation with 14 ng/μl Na_2_(PtCl_6_) (B), only some HEI-OC1 cells survived, whereas the other cells underwent necroptosis characterized by incomplete cell membranes and organelles as well as the loss of cytoplasmic components. At higher magnification of single surviving cells mitochondria could be seen in an increased amount, but were partly seen inside autophagolysosomes (C, arrowheads). The multilamellar vacuoles in the surroundings demonstrated the remnants of the digested mitochondria. Scale bars: 2 μm.

Ultrastructurally, the HEI-OC1 lost their cell integrity depending on the concentration of Na_2_(PtCl_6_). While in the untreated culture, the cell morphology remained almost intact even at the lowest tested concentration of 8 ng/μl compared to untreated cells according to Berger et al. (Fig 3A), only individual cells contained a larger amount of vesicles [22]. At higher Na_2_(PtCl_6_) concentrations, more cells lost their integrity because of swelling and disrupted membranes and could be seen only as cellular ghosts (Fig 3B). To evaluate the pathomechanism of PtCl_6_ ion intoxication, cell morphology was analyzed in more detail in cells exposed to higher concentrations of Na_2_(PtCl_6_) but still alive. Under this condition, the first pathological sign was an increased number of mitochondria. However, autophagosomes with mitochondria (mitophagy) were also detected, indicating a toxic mitochondrial defect (Fig 3C). The presence of a higher amount of multilamellar vacuoles with the indigestible membranous remnants of the mitochondria supported the repairing trail by autophagy in the fittest surviving cells in contact with a higher amount of Na_2_(PtCl_6_) within the limited incubation time of 48 h.

**Fig 4:**
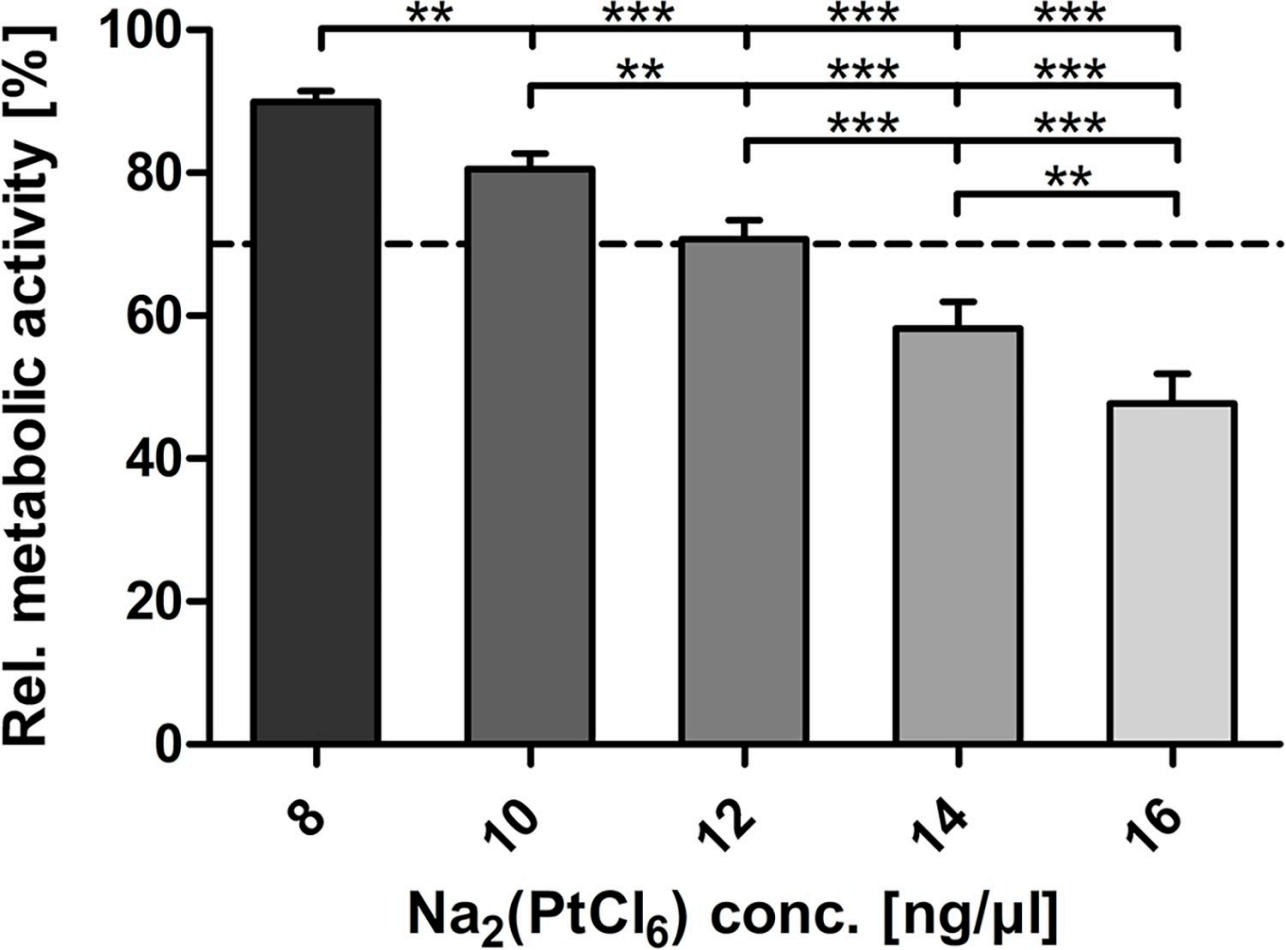
Determination of the oxidative activity of HEI-OC1 cells following exposure to Na_2_(PtCl_6_). The oxidative activity of HEI-OC1 cells exposed to varying concentrations of Na_2_(PtCl_6_) was examined by indirect reduction of resazurin in the presence of dehydrogenases to the highly fluorescent resorufin. The resulting fluorescence intensities, measured as fluorescence units (FU) after 48 h of cultivation, were relative to those of the untreated control group and calculated as percentages [%]. Each data point is presented as the mean ± SEM. Repeated measures ANOVA with Tukey’s multiple comparison test was performed for statistical assessment (*p ≤ 0.05). The dashed line represents the cytotoxicity limit (70%). A total of N = 10 independent experiments were conducted. Each experiment was performed in triplicate (n = 3).

The results of the relative quantification of the oxidative activities of the HEI-OC1 cells after exposure to increasing concentrations of Na_2_(PtCl_6_) reflected the approximately linear reduction in oxidative activity from 10 ng/μl (80.52% ± 2.2%) Na_2_(PtCl_6_), whereas 8 ng/μl (89.92% ± 1.52%) Na_2_(PtCl_6_) caused a moderate decrease in relation to the untreated cell group, as shown in Fig 4. Na_2_(PtCl_6_) concentrations below 12 ng/μl (70.71% ± 2.65%) met the criteria of ISO10993-5:2009, triggering cell death signaling in at least 30% of HEI-OC1 cells in relation to the untreated control. Administration of 14 ng/μl and 16 ng/μl Na_2_(PtCl_6_) resulted in severe decreases in oxidative activity of up to 60% (58.19% ± 3.77%) and 50% (47.74% ± 4.17%), respectively.

### The survival rate of SGC and neurite outgrowth of SGN were reduced following Na_2_(PtCl_6_) exposure in a concentration-dependent manner

**Fig 5:**
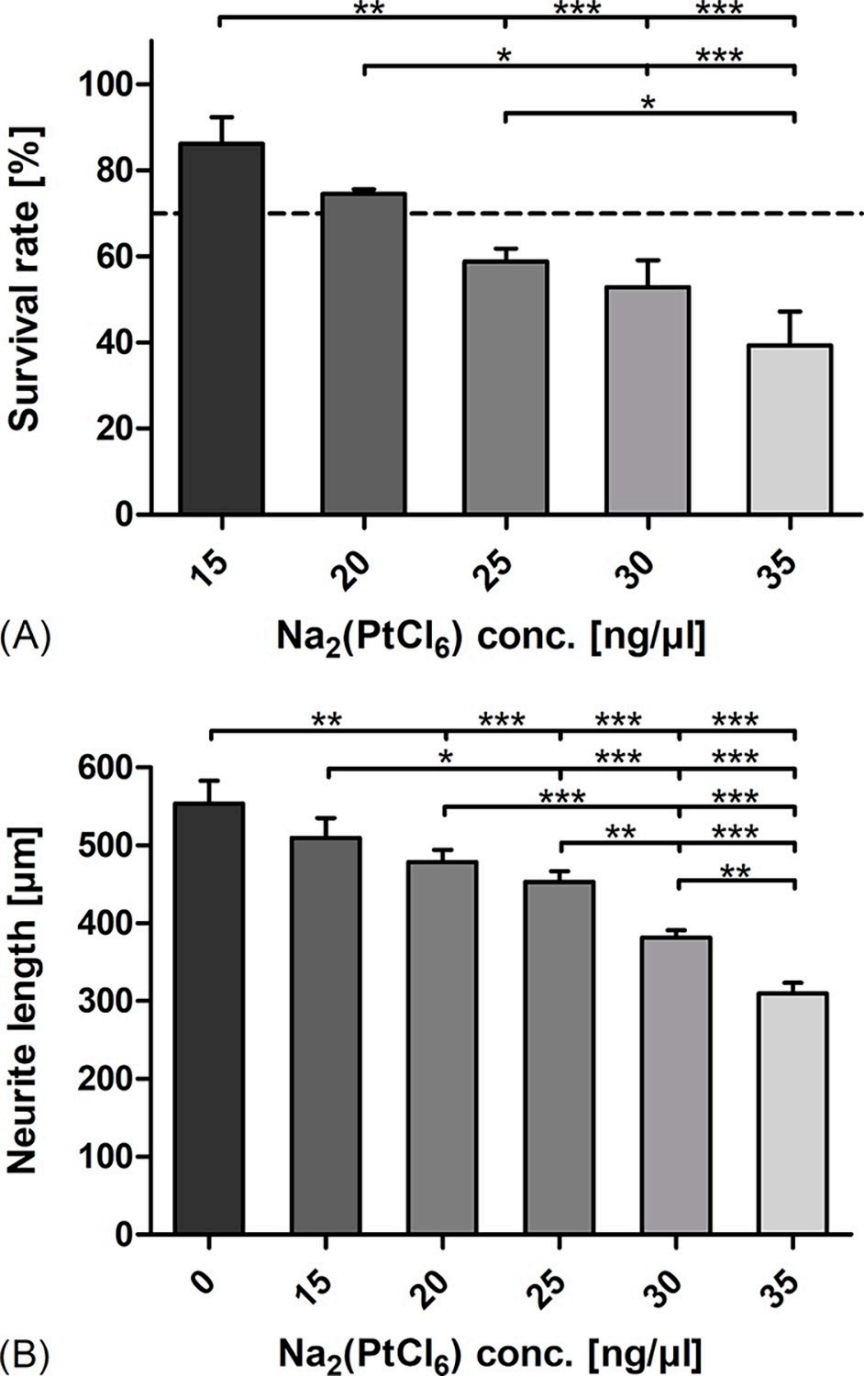
Determination of the survival rate and neurite outgrowth of the SGN following exposure to Na_2_(PtCl_6_). Relative quantification of the survival rate (A) and measurement of the neurite extensions (B) of the SGN cultivated in completed neuromedium supplied with 15–35 ng/μl Na_2_(PtCl_6_). Anti-neurofilament staining was used to visualize the soma and nerve fibers of the SGN. Each data point is presented as the mean ± SEM of the (A) percentage of labeled soma (N = 5, n = 3 of each Na_2_(PtCl_6_) concentration) relative to the control group without Na_2_(PtCl_6_) treatment and (B) length of neurites (N = 5, n = 15 neurons of each Na_2_(PtCl_6_) concentration). Repeated measures ANOVA with Tukey’s multiple comparison test was performed for statistical assessment. According to ISO 10993–5:2009, the dashed line in (A) represents the cytotoxicity limit (70%) [22].

In this study, the primary SGC culture model was used to characterize Na_2_(PtCl_6_)-induced cytotoxicity. The survival rates of SGN following supplementation with 15 ng/μl to 35 ng/μl Na_2_(PtCl_6_) showed a strong, almost linear reduction in neuronal survival. As shown in Fig 5A, 15 ng/μl Na_2_(PtCl_6_) slightly lowered the survival rate to 85% (86.09% ± 6.25%) in comparison to that of the control group without Na_2_(PtCl_6_) administration, which was defined as 100%. However, there was a tendency toward decreased neuronal survival in SGC culture assays containing 20 ng/μl Na_2_(PtCl_6_) (74.45% ± 1.15%). A further increase in the Na_2_(PtCl_6_) concentration from 25 ng/μl to 30 ng/μl and 35 ng/μl led to a strong decrease in the number of vital neurons, from 60% (58.84% ± 3.0%) to 50% (52.87% ± 6.19%) and 40% (39.24% ± 7.89%), respectively, relative to those in the untreated control group. Similar to the survival rates, the neurite length decreased in a concentration-dependent manner. Fig 5B shows highly significant reductions in neurite growth from an average of 554 nm (553.87 nm ± 29.6 nm) in the untreated control to 453 nm (453.45 nm ± 13.4 nm), 382 nm (381.67 nm ± 9.76 nm), 310 nm (309.75 nm ± 13.8 nm) in the samples exposed to 25 ng/μl, 30 ng/μl, 35 ng/μl Na_2_(PtCl_6_), respectively.

**Fig 6:**
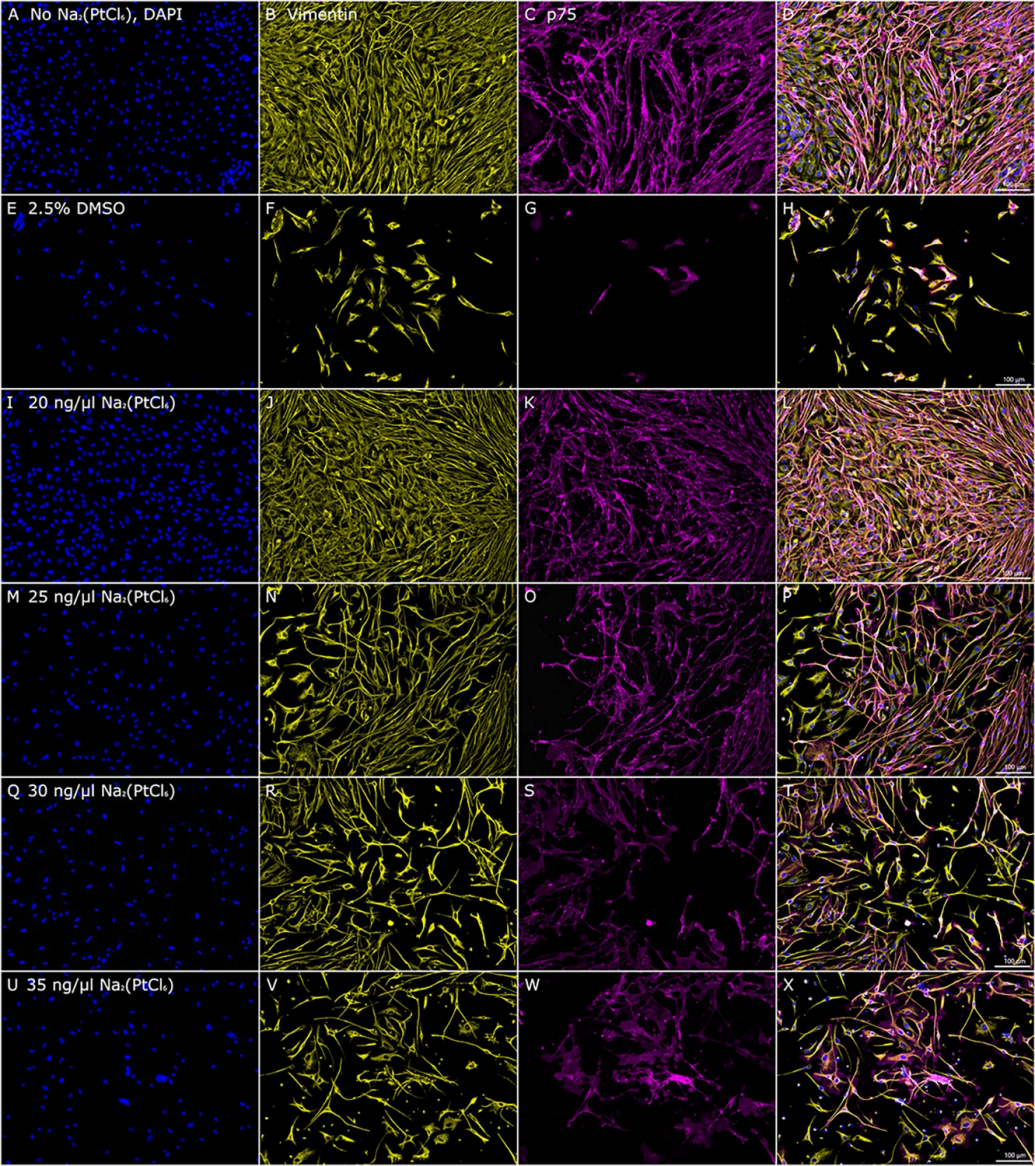
Representative immunofluorescence images of non-neuronal cells in SGC culture assays supplied with Na_2_(PtCl_6_). DAPI was used for nucleic staining (A, E, L, M, Q, U). Vim (labeled fibroblasts and glial cells, green, stained yellow in the images) and p75 antigen (binding to glial cells, red, stained magenta in the images) staining was found in the control group without Na_2_(PtCl_6_) incubation, indicating vital cells (A-D), whereas administration of 20 ng/μl (I-L), 25 ng/μl (M-P), 30 ng/μl (Q-T) and 35 ng/μl (U-X) resulted in detachment of the cells from the cell culture dish and retraction of the cell-cell contacts. SGC cultivation assays exposed to 2.5% DMSO served as a cell death control (E-H). The immunofluorescence images of the non-neuronal cells are representative of a total of N = 3 independent experiments. Each experiment was performed in triplicate (n = 3). Scale bars: 100 μm.

Evaluation of the cellular composition of the SGC culture model by using specific primary antibodies revealed widespread adhesion and tight cellular contacts of the fibroblasts and glial cells, as shown by the positive staining of vimentin (Vim) and p75 in both the untreated control (Fig 6A–6D) and the SGC culture assays supplemented with 20 ng/μl Na_2_(PtCl_6_) (Fig 6I–6L).

However, the application of 25 ng/μl Na_2_(PtCl_6_) resulted in decreased cell density and morphological changes (Fig 6M–6P), which were enhanced after exposure to higher Na_2_(PtCl_6_) concentrations of up to 35 ng/μl (Fig 6Q–6X). Additionally, the detachment of fibroblasts and glial cells from the cell culture plate surface and the formation of tiny spherical cells were observed in a dose-dependent manner (Fig 6Q–6X). Staining of the SGC exposed to DMSO revealed a greatly reduced number of cells of both types investigated in this study (Fig 6E–6H).

### Dose-dependent loss of hair cells in the OC_ex_ following organotypic cultivation in the presence of Na_2_(PtCl_6_)

**Fig 7:**
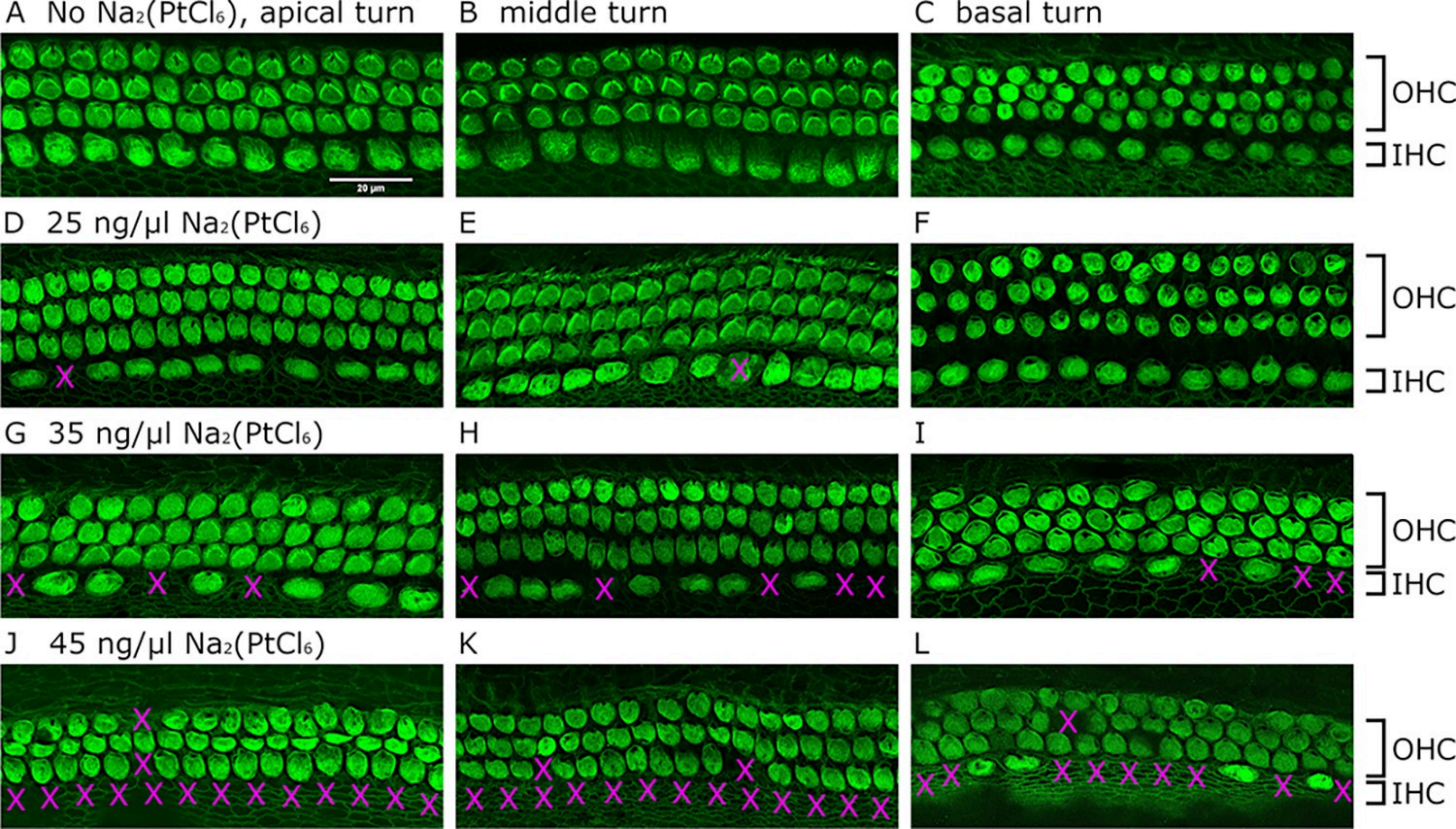
Representative fluorescence images of OC_ex_ hair cells following organotypic cultivation in the presence of Na_2_(PtCl_6_). Confocal laser scanning microscopy images of the soma of hair cells without any Na_2_(PtCl_6_) treatment showed intact rows of the IHC and OHC in all cochlear parts (A-C). In contrast, the application of 25 ng/μl (D-F), 35 ng/μl (G-I) and 45 ng/μl (J-L) Na_2_(PtCl_6_) resulted in a dose-dependent decrease in the number of IHC in all cochlear turns, followed by the decrease in the number of OHC at the highest Na_2_(PtCl_6_) concentration used in this study (J-L). Magenta crosses indicate the loss of hair cells. The fluorescence images of the OC_ex_ hair cells are representative of a total of N = 3 independent experiments. Each experiment was performed in triplicate (n = 3). Scale bar for all images: 20 μm.

In addition to the inner ear-related cell line, OC were extracted from neonatal rats for organotypic cultivation in the presence of Na_2_(PtCl_6_) at concentrations between 25 ng/μl and 45 ng/μl. As shown in Fig 7A–7C, both the inner and outer hair cells (IHC and OHC) appeared intact in representative images of the apical, middle and basal turns of the OC_ex_ samples without Na_2_(PtCl_6_) treatment, whereas incubation of the OC_ex_ samples with 25 ng/μl Na_2_(PtCl_6_) induced the death of a few IHC in the apical and middle turns of the OC_ex_ (Fig 7D and 7E), but not in the basal turn (Fig 7F). Higher concentrations of 35 ng/μl Na_2_(PtCl_6_) led to increased numbers of missing IHC not only in the apical and middle turns but also in the basal turns (Fig 7G–7I). Exposure to 45 ng/μl Na_2_(PtCl_6_) resulted in nearly complete IHC loss in all turns, as demonstrated in Fig 7J–7L. Additionally, compared to untreated OC_ex_, a lack of OHC could be observed and cell morphology was altered.

## Discussion

This study was the first to investigate the cytotoxic effects of Na_2_(PtCl_6_) on primary inner ear sensory cells and neurons as well as on an inner ear-related cell line. This Pt(IV) compound does not belong to the class of antineoplastic drugs. Similar to our data obtained from the non-inner ear-related cell lines NIH 3T3 and SH-SY5Y exposed to ionic Pt generated by electrochemical stimulation of the human CI electrode [20], a concentration-dependent cytotoxicity of Na_2_(PtCl_6_) in all examined cell culture models was found, confirming the authors’ established hypothesis.

However, this study showed that susceptibility to Na_2_(PtCl_6_)-induced cell death may also depend on the cell type and tissue structure: signs of oxidative stress in the HEI-OC1 cell line were already observed following the administration of 10 ng/μl Na_2_(PtCl_6_), whereas concentrations of 20 ng/μl and 25 ng/μl were required to trigger cell death in the SGC and OC_ex_, respectively. Furthermore, 12 ng/μl Na_2_(PtCl_6_) increased cytotoxicity in the organ of the Corti cell line within 48 h of cultivation, resulting in a decrease in oxidative activity of at least 30% of the HEI-OC1 cells compared to cells without Na_2_(PtCl_6_) treatment. According to these data, the ultrastructurally detected mitophagy can be interpreted as repair mechanism of the HEI-OC1 cells to Na_2_(PtCl_6_)-induced toxicity on mitochondria. Furthermore, dysfunctional mitochondria cause ATP depletion, leading to cell death. These results suggest that Na_2_(PtCl_6_) induces a reduction in dehydrogenases, which are also highly concentrated in the mitochondria. Consequently, cellular survival depends on the capacity of the remaining mitochondria to induce regeneration processes. Otherwise, cells undergo necroptosis, as shown by swelling of all cell organelles and loss of membrane integrity [26, 27]. In addition, the cellular loss observed in the microscopic images indicated the loss of the cytoskeleton as well as cell-cell junctions and cell-matrix adhesion points, changing the morphology from flat-shaped cells with broad lamellipodia to spherical cells with DNA intercalation of EthD. Finally, exposure to 16 ng/μl Na_2_(PtCl_6_) resulted in cell death in more than half of the HEI-OC1 cells.

Compared to the organ of the Corti cell line, primary cells such as dissociated SGC appear to be less sensitive to Pt compounds: severe oxidative stress occurred at Na_2_(PtCl_6_) concentrations of approximately 25 ng/μl, when the number of surviving neurons was significantly reduced to below 60% compared to the untreated control group. The difference in susceptibility to Na_2_(PtCl_6_) in both cell culture models may be related to their cellular structure. Whereas HEI-OC1 cells represent a uniform cell line, the primary neurons of both the SGC and OC_ex_ are surrounded by fibroblasts and glial cells, including Schwann cells and satellite glial cells. These non-neuronal cells have been shown to contribute to neuronal survival and regeneration mechanisms by myelination of axons, enabling trophic support—particularly by brain-derived neurotrophic factor—and the expression of extracellular matrix proteins [28–30]. In particular, satellite glial cells connected by gap junctions are thought to maintain and protect auditory neurons via connexin 43-mediated signaling [29]. According to a previous study reporting SGN loss in the context of retracted adhesion of surrounding cells [31], the Na_2_(PtCl_6_)-induced reduction in neuronal survival and neurite extension must be considered in the context of fibroblast and glial cell morphological changes and their subsequent loss. However, compared to the HEI-OC1 cell culture model, an approximately twofold Na_2_(PtCl_6_) concentration was required for cell death induction in the SGC, as defined by ISO 10993–5:2009, published previously [22].

We suggest that Na_2_(PtCl_6_) resistance may be related not only to the distinct diversity of the primary cell culture, but also to the trophic and protective impact of glial cells on the SGN. Moreover, in comparison to the organ of the Corti cell line HEI-OC1, strong resistance against Na_2_(PtCl_6_)-induced stress was observed in the organotypic cultivation of the OC_ex_. Only a few IHC underwent cell death following exposure to 25 ng/μl Na_2_(PtCl_6_), whereas higher Na_2_(PtCl_6_) concentrations up to 45 ng/μl Na_2_(PtCl_6_) resulted in severe IHC and OHC loss. It may be assumed that the OC_ex_ enables the control of various protection and repair mechanisms triggered by glia, such as supporting cells, as several types of phalangeal and pillar cells have been described [32, 33]. As reviewed by Waissbluth et al., supporting cells are widely connected to hair cells mediating functions in communication, homeostasis, trophic support and regeneration, and in comparison to hair cells, supporting cells are more robust upon stress [33]. Previous *in vitro* and *in vivo* studies in murine utricles and cochleae reported the prevention of cisplatin-induced hair cell death following heat shock protein (HSP) 70 expression [34, 35]. May and colleagues (2013) demonstrated HSP-70 expression in glia-like supporting cells of the utricles. By mediating cell survival by supporting the refolding of denatured proteins as well as impeding the protein aggregation, HSP-70 may protect not only OC, but also SG [36, 37].

Since the effects of hexachloroplatinate on inner ear tissues are poorly described thus far, only datasets evaluated with antineoplastic substances such as cisplatin can be used to contrast with those obtained from Na_2_(PtCl_6_) experiments to evaluate potential ototoxicity in the inner ear. Our study confirmed the results of numerous cisplatin studies: exposing HEI-OC1 cells to 25–30 μM cisplatin resulted in a cell viability of approximately 50%, which corresponded well to the 14–16 ng/μl (25–28.5 μM) Na_2_(PtCl_6_) observed in our experiments [38–41]. Whereas the half-lethal concentrations of cisplatin and Na_2_(PtCl_6_) in the auditory cell line were comparable, cisplatin cytotoxicity varied between 6.6 μM and 16.7 μM in the SGC culture models, and oxidative stress was reported at lower concentrations [42, 43] than those observed in SGC cultures supplied with Na_2_(PtCl_6_). In comparison to our results on the effects of Na_2_(PtCl_6_) in OC_ex_, hair cell death was reported following cisplatin exposure at concentrations between 2 μM, 16 μM and 40 μM, especially in IHC [42, 44, 45], while OHC were lost first, followed by IHC damage depending on the dose [38, 45] and cultivation period [44]. Additionally, the loss of OHC and IHC was mainly demonstrated in the middle and basal turns but not in the apical turn [45]. Interestingly, Na_2_(PtCl_6_) exposure, even at high concentrations (44.5 μM ≙ 25 ng/μl, 80 μM ≙ 45 ng/μl) evidenced not only less toxic effects in comparison to cisplatin, but also triggered cell death in IHC first and only subsequently in OHC. Additionally, gaps in the IHC and OHC rows were found in the apical and middle turns rather than in the basal turns. Basal turns have more supporting cells, which are likely responsible for the protective effect on the sensory cells. Differences in cytotoxicity, especially in inner ear primary cells and tissues, as well as preferences in tissue targets may be related to the molecular structures of both Pt compounds: in contrast to cisplatin, which is surrounded by two amino and chloride ligands in a cis-configuration forming a square planar molecule, the Pt core in Na_2_(PtCl_6_) assembles six chloride ligands presenting a spherical structure with higher molecular size. As reported in previous studies, cisplatin is transformed to aquo complexes by exchanging chlorine ligands for water molecules once it enters the cytoplasm, enabling irreversible binding to nucleic acids and proteins [46, 47]. It remains to be clarified to what extent Na_2_(PtCl_6_) can form aquo complexes and also induce cell death by disrupting DNA replication and transcription or by interacting with the active site of enzymes. The question arises as to how the oxidation state and the number of coordinated halogens affect cell penetration and cell damage potential. A recent study investigated the relationship between oxidation state and skin permeation and demonstrated a higher potential of potassium hexachloro(IV) platinate to overcome skin barrier rejection compared to tetrachloro(II) platinate [48]. In addition, using a mouse model of hypersensitivity, it was found that cisplatin did not induce an immediate respiratory response in the range of increased chlorine ligands, in contrast to ammonium tetrachloro(II)platinate and hexachloro(IV)platinate, respectively [49–51]. However, these findings do not reflect the results of our study, which suggest that other cellular mechanisms influence the potency of halogenated Pt compounds to induce cell damage. Furthermore, it must be considered that corrosion of Pt electrode contacts involves the formation of Pt complexes other than hexachloro(IV)platinate alone. The interaction of corrosion products with target cells in the highly complex inner ear environment remains a challenge.

Cisplatin and other antineoplastic compounds were described to be involved not only in apoptosis but also in necroptosis and autophagy [52, 53]. Hence, Na_2_(PtCl_6_) may be involved in similar stress pathways, which needs to be investigated further. To mediate cytotoxic pathways, Pt compounds—either cisplatin or Na_2_(PtCl_6_)—must be internalized by target cells. There is increasing evidence that cellular uptake is conducted by the active transfer of ions and molecules using membrane transporters such as organic cation transporter 2 (OCT2) and copper transporter (Ctr1) since antineoplastic Pt compounds are highly polarized molecules that are unable to diffuse through the phospholipid plasma membrane [32, 52, 54]. As reviewed by Waissbluth et al., OCT2 and Ctr1 expression has been demonstrated in almost all cochlear tissues [33]. It may be speculated that water soluble Pt compounds such as Na_2_(PtCl_6_) are actively transferred into the cytoplasm by membrane transporters, as could be shown for cisplatin.

So far, there are no data on the *in vivo* toxicity of neural implant corrosion products in humans or how they are distributed in targeted tissues. Depending on the stimulation conditions *in vitro* release of Pt ions from electrode contacts [8, 55] and Pt discs [16] has been found at levels ranging from 5 pg/μl to 1 ng/μl [8, 55] as well 4.5 μg/cm^2^ at its maximum [16]. To date, in vivo quantification of Pt release from the cochlea has been performed in cats and guinea pigs [12, 13]. Depending on charge densities and other stimulation parameters, trace amounts of Pt were found in guinea pigs between 93–147 ng/mg cochlea after 28 d of chronic stimulation [12, 13]. In contrast, electrode corrosion in cats was measured in the range of 1.92–3.36 μg/mg cochlea after 6 months of electrical stimulation. It was found that chronic stimulation resulted in a strong tissue response, increased Pt corrosion and trapping of Pt particles in the tissue capsule within the electrode trace. Although the stimulation parameters were set above those used clinically, no stimulus-induced loss of auditory neurons and neuronal function was found [12, 13]. On the one hand, the results described in the in vivo studies may be related to the detection of solid Pt particles rather than ionic Pt. On the other hand, it must be considered that the diffusion of Pt ions within the cochlea via perilymphatic fluids may reduce their concentration and therefore the potential for cellular damage. However, long-term electrical stimulation over several years may support the accumulation and potentiate adverse effects of the corrosion products on the sensory tissues at the Pt/perilymphatic fluid interface, and these effects remain to be elucidated. It is of clinical interest to limit the corrosion of the electrode contacts and to counteract the resulting tissue damage, since hearing impaired people are expected to benefit from cochlea implants (CIs) throughout their lives.

## Conclusion

This study evaluated the cytotoxicity of a Pt(IV) compound, Na_2_(PtCl_6_), which is suspected to represent one of the corrosion products of the nerve-electrode interface of CIs. For the first time, inner ear-related cells were used as cell culture models: the HEI-OC1 cell line, primary rat SGC and the OC_ex_. In all cell and tissue culture models, Na_2_(PtCl_6_)-induced oxidative stress, resulting in cell death in a concentration-dependent manner. In particular, the IHC of the OC_ex_ disappeared first, followed by the OHC at higher concentrations. In contrast to those in the HEI-OC1 cell line, the resistance to Na_2_(PtCl_6_)-induced oxidative stress in the SGC and OC_ex_ cultivation models may be related to trophic support and regeneration mechanisms triggered by non-neuronal cells such as Schwann, satellite glial and supporting cells. On the other hand, stress-induced morphological changes in non-neuronal cells and their subsequent loss are associated with a decrease in neuronal survival, neurite outgrowth and hair cells. Contrary to cell culture assays in which cells were exposed to antineoplastic Pt compounds, with cisplatin being the best characterized Pt compound, cytotoxic effects were observed in SGC and OC_ex_ culture models at higher Na_2_(PtCl_6_) concentrations. It appears that the molecular structure of Pt compounds allows them to enter target cells and interact with metabolic processes. The active transport mechanisms by which corrosion products are internalized and which cell death signaling pathways are triggered by Pt compounds remain to be clarified. Finally, it is of clinical interest not only to understand the mechanisms of corrosion and the interactions of the corrosion products with the cochlear targets, but also to investigate potential inhibitors that support cell repair mechanisms.

## Supporting information

S1 Dataset Minimal dataset. Minimal underlying dataset of the study.
(PDF)

S1 Fig Composite image from Fig 6 in high resolution. Representative SGC culture assay with no Na_2_(PtCl_6_) as reference.
(TIF)

S2 Fig Composite image from Fig 6 in high resolution. Representative SGC culture assay with 20 ng/μl Na_2_(PtCl_6_).
(TIF)

S3 Fig Composite image from Fig 6 in high resolution. Representative SGC culture assay with 25 ng/μl Na_2_(PtCl_6_).
(TIF)

S4 Fig Composite image from Fig 6 in high resolution. Representative SGC culture assay with 30 ng/μl Na_2_(PtCl_6_).
(TIF)

S5 Fig Composite image from Fig 6 in high resolution. Representative SGC culture assay with 35 ng/μl Na_2_(PtCl_6_).
(TIF)

S6 Fig Composite image from Fig 6 in high resolution. Representative SGC culture assay with 2.5% DMSO as cell death control.
(TIF)

## Abbreviations

HEPES: 4-(2-hydroxyethyl)-1-piperazineethanesulfonic acid
ANOVA: analysis of variance
bFGF: basic fibroblast growth factor
BSA: bovine serum albumin
Calcein AM: Calcein acetoxymethyl
CI: cochlear implant
Cis: cochlear implants
Ctr 1: copper transporter
DNA: deoxyribonucleic acid
DMEM: Dulbeccos modified Eagle medium
DMSO: dimethyl sulfoxide
ER: endoplasmic reticulum
EthD: Ethidium homodimer III
FCS: fetal calf serum
FU: fluorescence units
HBSS: Hank’s balanced salt solution
HEI-OC1: Hearing-Ear-Institute-Organ of Corti cell line
HSP: heat shock protein
SH-SY5Y: human neuroblastoma cell line
EGF: human recombinant epidermal growth factor
IHC: inner hair cells
NAC: N-acetyl-L-cysteine
NIH 3T3: National Institutes of Health-Swiss mouse embryonic fibroblast cell line
NF: neurofilament
OCT2: organic cation transporter 2
OC: organ of Corti
OC_ex,_: organ of Corti explants
OHC: outer hair cells
PBS: phosphate buffered saline
Pt: platinum
Na_2_(PtCl_6_): sodium hexachloroplatinate (IV)
p75: p75 neurotrophin receptor
SG: spiral ganglia
SGC: spiral ganglion cells
SGN: spiral ganglion neurons
SEM: standard error of the mean
TOF-SIMS: time of flight secondary ion mass spectrometry
Vim: vimentin
