# Supplementary material for: Induction of cell death by sodium hexachloroplatinate (IV) in the HEI-OC1 cell line, primary rat spiral ganglion cells and rat organ of Corti explants
Source: PLoS One. 2024 Jul 26;19(7):e0307973. doi: 10.1371/journal.pone.0307973 (PMC11280268; doi:10.1371/journal.pone.0307973)
Supplement: S1 Dataset — Minimal underlying dataset of the study. (PDF) [file pone.0307973.s001.pdf]

MINIMAL DATA SET

Survival rate of SG neurons following $Na_2(PtCl_6)$ incubation

15 ng/µl			20 ng/µl			25 ng/µl			30 ng/µl			35 ng/µl		
58,72	95,41	161,47	50,46	97,25	66,97	55,96	63,30	46,79	44,04	41,28	64,22	55,05	23,85	11,01
105,44	120,39	61,72	91,61	77,21	54,24	78,87	52,31	60,06	68,63	63,38	56,18	68,91	44,00	38,75
77,70	57,43	83,78	84,46	97,30	45,95	53,38	73,65	41,22	25,68	32,43	37,16	18,92	12,16	12,16
75,38	69,74	78,46	77,95	68,21	71,28	34,87	61,03	57,95	47,69	55,38	54,36	42,56	47,69	35,38
79,35	89,10	77,26	71,69	73,78	88,40	70,30	71,69	61,25	76,57	56,38	69,61	57,77	60,56	59,86

Data are presented as normalized against the untreated cells.

Neurite outgrowth of SG neurons following $Na_2(PtCl_6)$ incubation

PK														
551,22	447,43	493,35	427,6	574,83	376,62	589,82	360,76	374,11	423,43	579,01	341,33	359,78	336,63	710,52
448,5	480,17	570,41	445,17	590,09	473,39	523,04	521,68	732,08	540,45	452,53	590,09	645,82	789,47	668,85
359,99	569,97	390,56	449,96	400,18	717,18	568,81	480,36	445,91	334,82	774,06	466,07	443,86	686,48	595,55
449,69	469,17	689,21	470,55	486,38	695,1	603	567,97	548,44	508,28	761,35	612,36	829,34	546,67	881,83
503,38	546,45	535,72	893,55	790,3	579,98	896,99	546,76	705,2	626	681,92	667,15	440,93	449,9	454,43

15 ng/µl														
505,89	362,07	287,84	439,52	423,04	414,44	299,19	519,85	550,99	382,34	343,52	543,36	437,51	590,64	628,23
409,3	599,17	801,05	414,64	427,46	445,63	480	338,05	533,01	361,12	533,74	462	513,12	562,58	305,86
640,66	505,96	469,66	463	532,73	542,36	608,51	400,22	339,77	424,68	382,49	657,78	391,92	481,46	448,14
648,67	545,59	400,58	381,96	346,06	673,22	507,79	601,72	554,9	521,58	607,39	526,72	487,44	790,1	537,36
555,23	593,35	538,58	559,34	537,48	678,73	357,32	677,29	685,16	546,75	499,88	606,52	788,28	586,73	680,1

20 ng/µl														
387,83	411,48	550,18	407,09	367,37	352,45	768,75	768,38	510,73	251,6	344,86	398,77	377,15	346,68	289,15
519,65	342,06	524,31	349,05	512,89	265,26	464,98	677,14	360,53	409,41	367,71	512,28	448,68	376,38	716,82
499,21	471,2	382,4	436,8	436,99	492,83	422,98	379,62	521,37	500,88	324,99	684,21	521,64	519,85	584,61
495,87	411,74	609,58	478,86	488,58	514,54	492,5	604,11	555,43	402,71	505,04	414,46	719,12	337,63	489,62
422,33	530,18	379,52	543,7	595,57	360,22	509,63	381,14	713,94	574,95	534,97	573,01	703,11	481,81	526,23

25 ng/µl														
375,88	419,25	510,34	501,78	621,4	432,72	343,89	414,81	472,25	474,03	283,76	413,98	345,12	343,8	378,81
417,82	464,59	397,13	556,61	410,96	430,29	464,06	573,21	355,06	322,66	448,33	509,73	354,49	401,44	416,24
294,95	398,73	516,27	530	492,51	395,20	527,39	580,53	459,66	440,03	412,33	436,96	346,92	334,72	444,66
362,35	417,62	521,15	337,80	433,38	403,84	479,49	610,79	468,32	693,88	307,86	548,73	616,55	413,24	531,48
622,39	480,86	555,52	445,93	354,79	698,65	374,85	558,39	532,03	360,39	709,64	396,86	370,11	351,66	585,10

30 ng/µl														
309,47	563,74	370,3	263,18	270,75	266,81	287,7	343,77	322,58	485,37	506,53	339,85	293,21	632,36	344,79
502,53	324,18	386,68	382,02	466,28	303,72	334,43	329,33	318,27	455,92	404,86	397,5	415,72	422,74	397,71
148,49	250,72	316,64	298,55	293,26	441,33	348,46	379,05	319,38	355,66	351,49	227,71	651,40	640,19	470,72
223,73	613,21	261,85	202,38	383,86	198,23	695,96	242,04	406,36	393,34	345,12	379,88	456,81	385,14	258,88
451,18	382,72	289,01	420,41	324,20	260,44	473,33	549,70	508,91	445,07	460,49	496,70	501,92	272,08	406,71

35 ng/µl														
231,94	274,44	258,64	346,89	236,52	319,84	205,18	393,28	378,12	219,32	462,46	305,13	93,57	147,16	157,72
241,4	370,75	716,09	285,5	266,58	235,88	289,88	362,98	411,59	442,12	244,46	231,61	318,28	262,83	357,9
260,98	204,41	330,71	378,49	338,42	259,55	338,37	429,89	265,33	188,90	239,90	364,96	287,67	242,29	143,01
466,13	224,20	359,32	198,12	355,68	289,37	305,49	453,36	252,9	511,63	286,88	264,68	357,78	330,05	289,23
292,44	244,02	389,44	313,86	370,4	554,22	314,22	401,75	302,84	348,5	251,17	304,08	283,9	344,28	230,41

Cell viability assay of HEI-OC1 cells following $Na_2(PtCl_6)$ incubation

8 ng/µl			10 ng/µl			12 ng/µl			14 ng/µl			16 ng/µl		
94,46	85,83	78,27	73,96	73,75	68,45	65,26	62,97	63,90	52,21	45,83	47,95	37,67	28,82	37,26
97,50	86,33	81,75	90,54	83,30	76,57	68,43	63,80	65,15	54,31	44,26	60,57	40,57	39,58	37,85
97,73	100,07	91,68	98,62	92,67	91,33	91,83	83,21	84,23	87,27	82,96	81,51	72,20	73,29	69,47
84,75	86,85	83,69	85,68	80,37	81,22	80,53	72,04	72,60	61,27	54,20	60,37	48,96	50,30	41,11
104,70	82,75	98,35	90,81	69,47	74,10	76,44	59,23	66,63	54,83	47,14	47,86	40,24	37,61	39,49
87,59	94,46	83,21	71,55	73,97	77,67	64,36	57,83	52,30	38,07	45,86	44,42	30,56	29,18	28,93
100,77	89,52	93,40	76,59	71,34	67,08	78,39	59,85	56,43	54,42	55,06	45,02	47,63	46,51	35,76
89,87	92,57	101,70	87,52	79,08	80,74	90,99	71,43	77,42	68,70	64,19	63,58	58,97	49,61	55,10
81,72	78,21	93,12	85,55	78,53	96,27	70,23	69,15	76,48	56,57	65,80	80,80	57,60	66,44	60,56
79,30	91,04	86,35	70,29	78,08	90,35	67,73	76,76	75,57	54,83	55,71	70,20	62,76	53,54	54,51

Data are presented as normalized against the untreated cells.
