# Supplementary figures and images for: Induction of cell death by sodium hexachloroplatinate (IV) in the HEI-OC1 cell line, primary rat spiral ganglion cells and rat organ of Corti explants
Source: PLoS One. 2024 Jul 26;19(7):e0307973. doi: 10.1371/journal.pone.0307973 (PMC11280268; doi:10.1371/journal.pone.0307973)

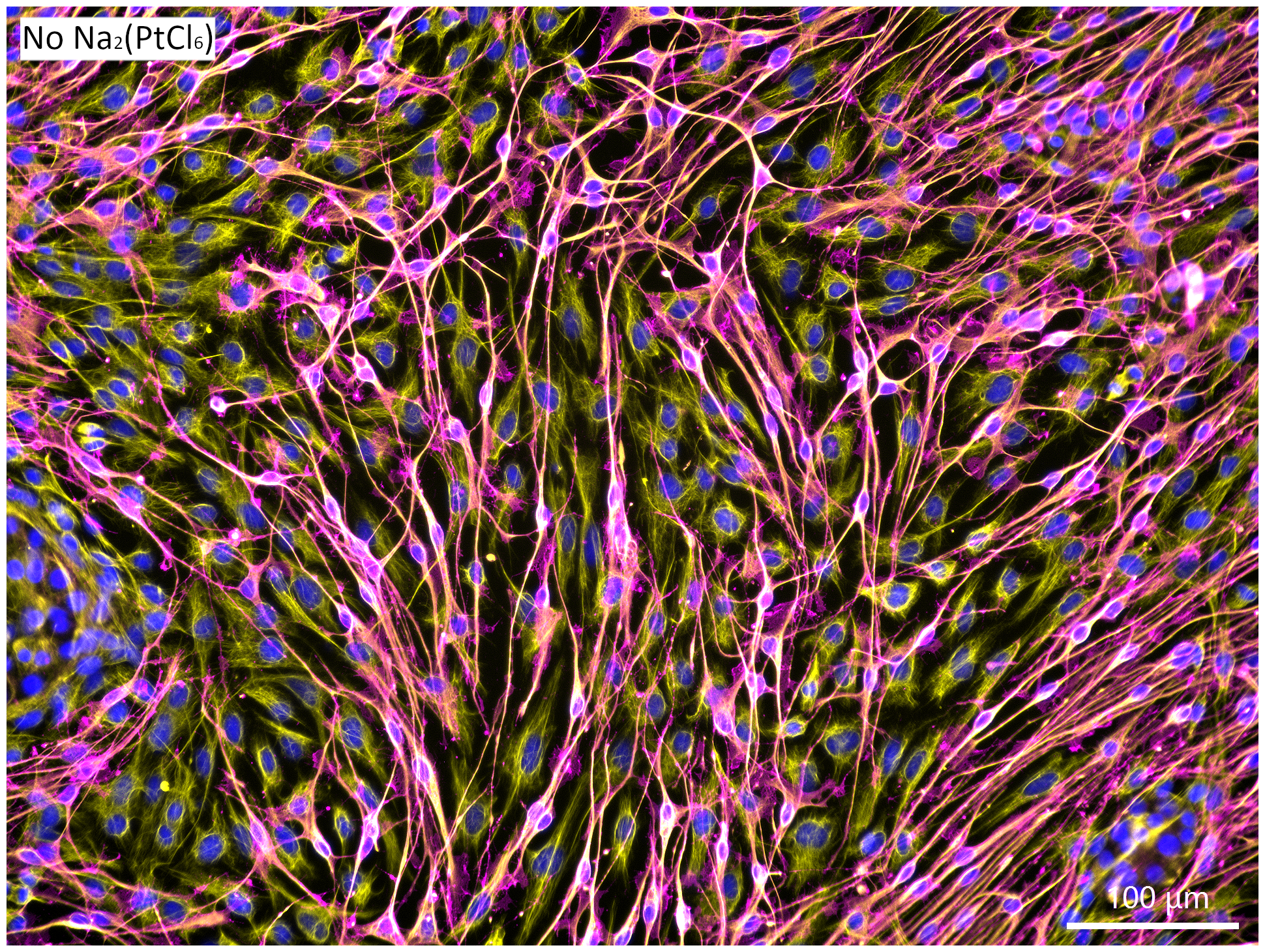

Supplement: S1 Fig — Representative SGC culture assay with no Na2(PtCl6) as reference. (TIF) [file pone.0307973.s002.tif]

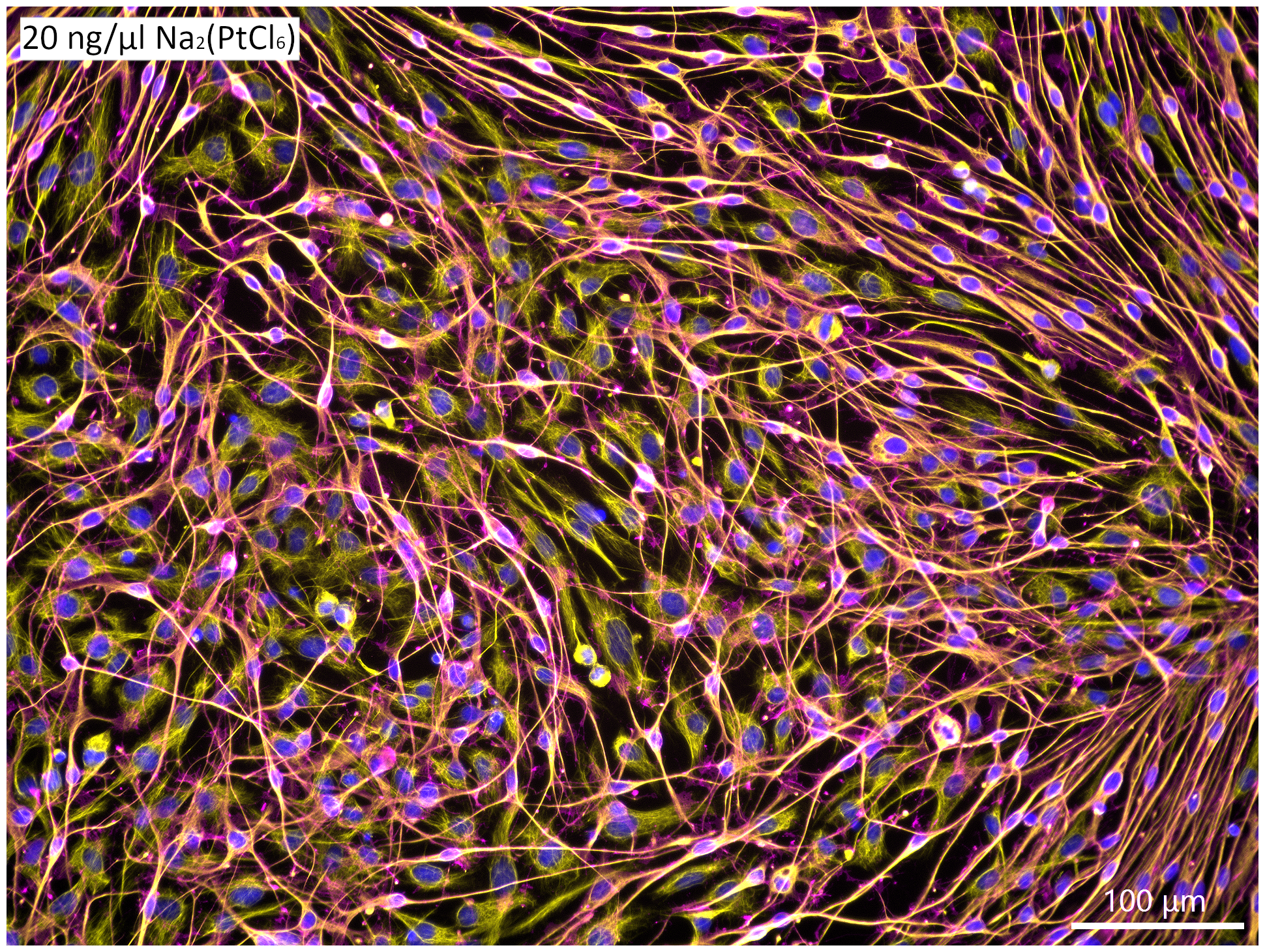

Supplement: S2 Fig — Representative SGC culture assay with 20 ng/μl Na2(PtCl6). (TIF) [file pone.0307973.s003.tif]

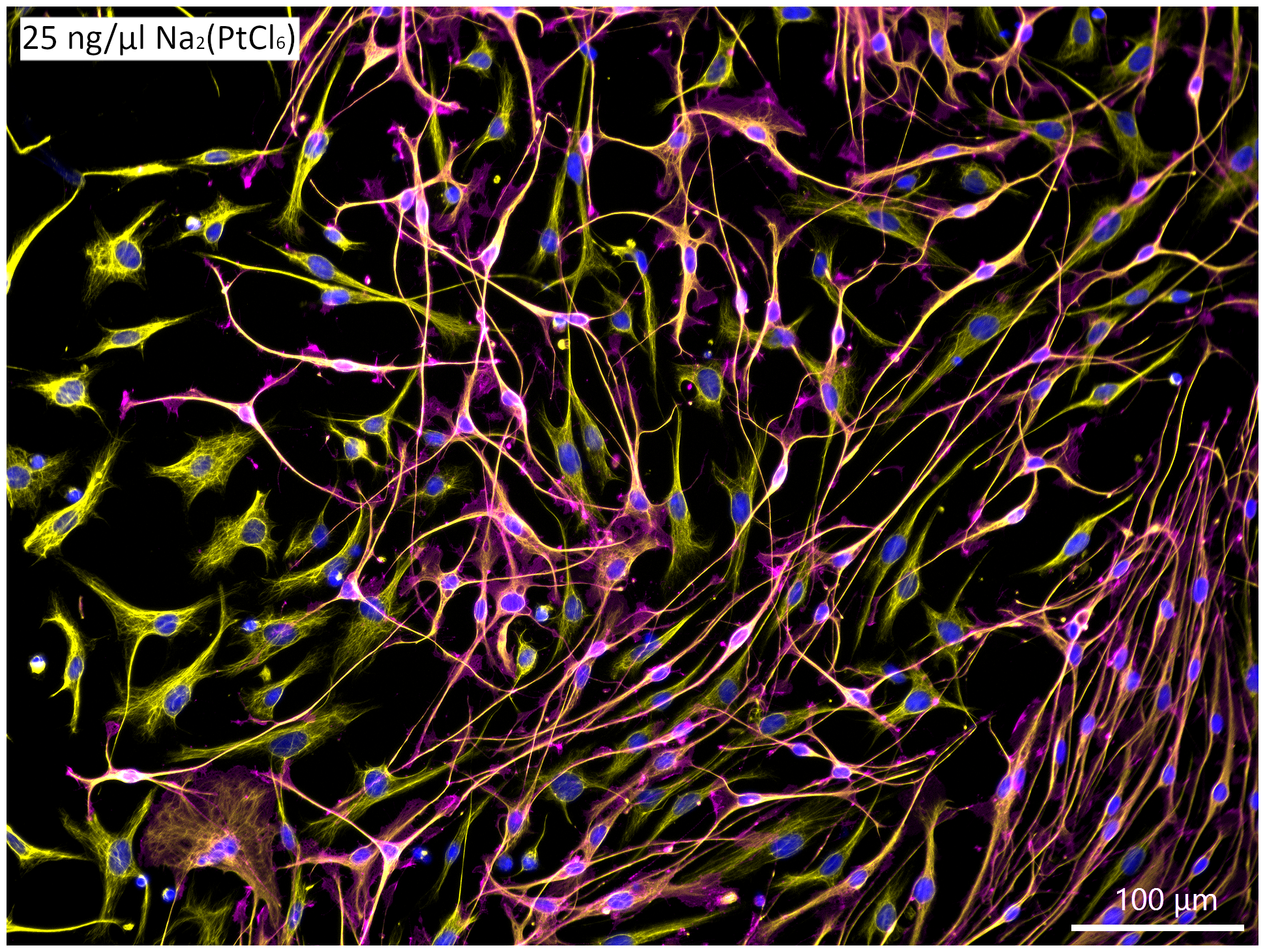

Supplement: S3 Fig — Representative SGC culture assay with 25 ng/μl Na2(PtCl6). (TIF) [file pone.0307973.s004.tif]

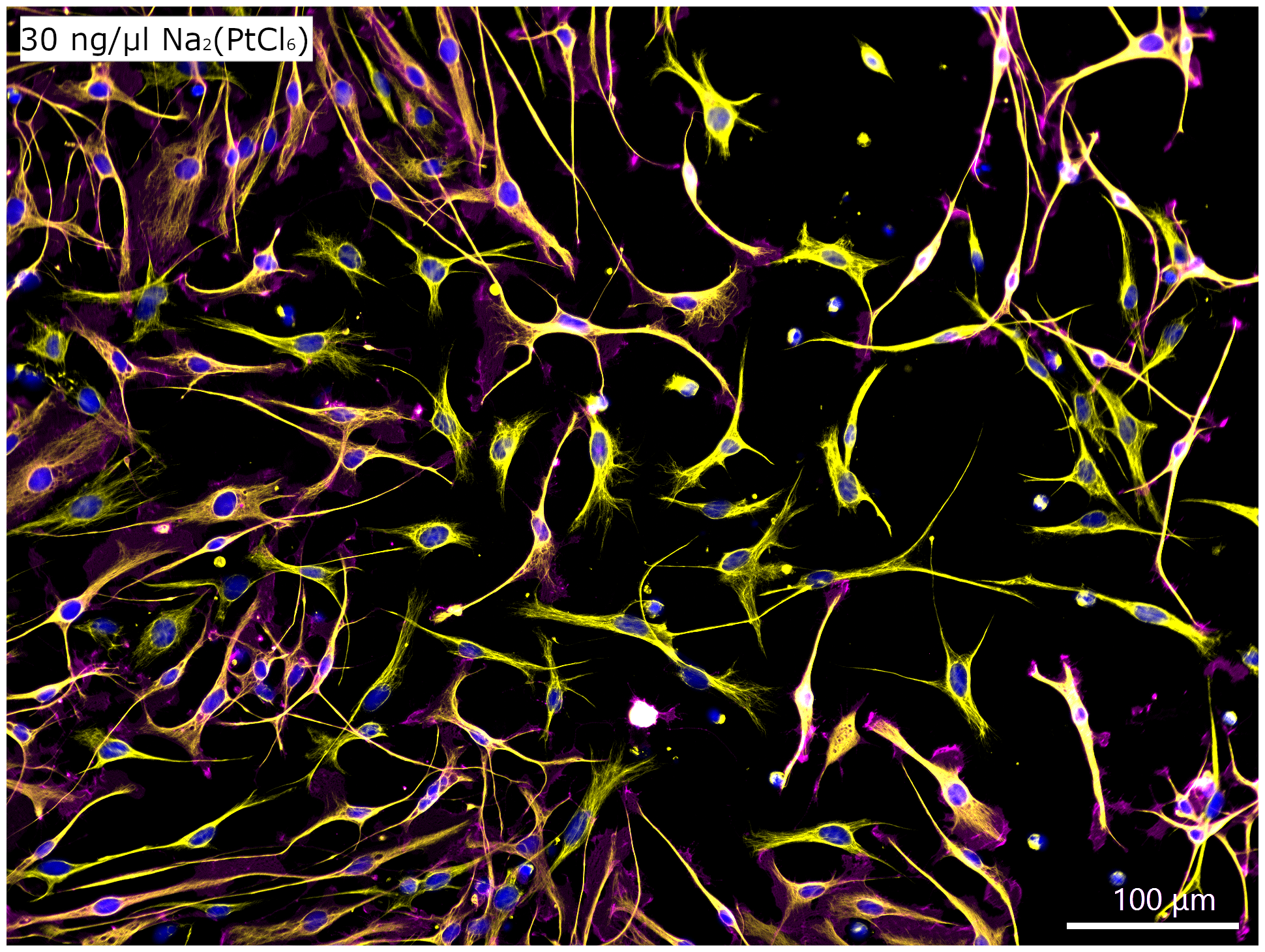

Supplement: S4 Fig — Representative SGC culture assay with 30 ng/μl Na2(PtCl6). (TIF) [file pone.0307973.s005.tif]

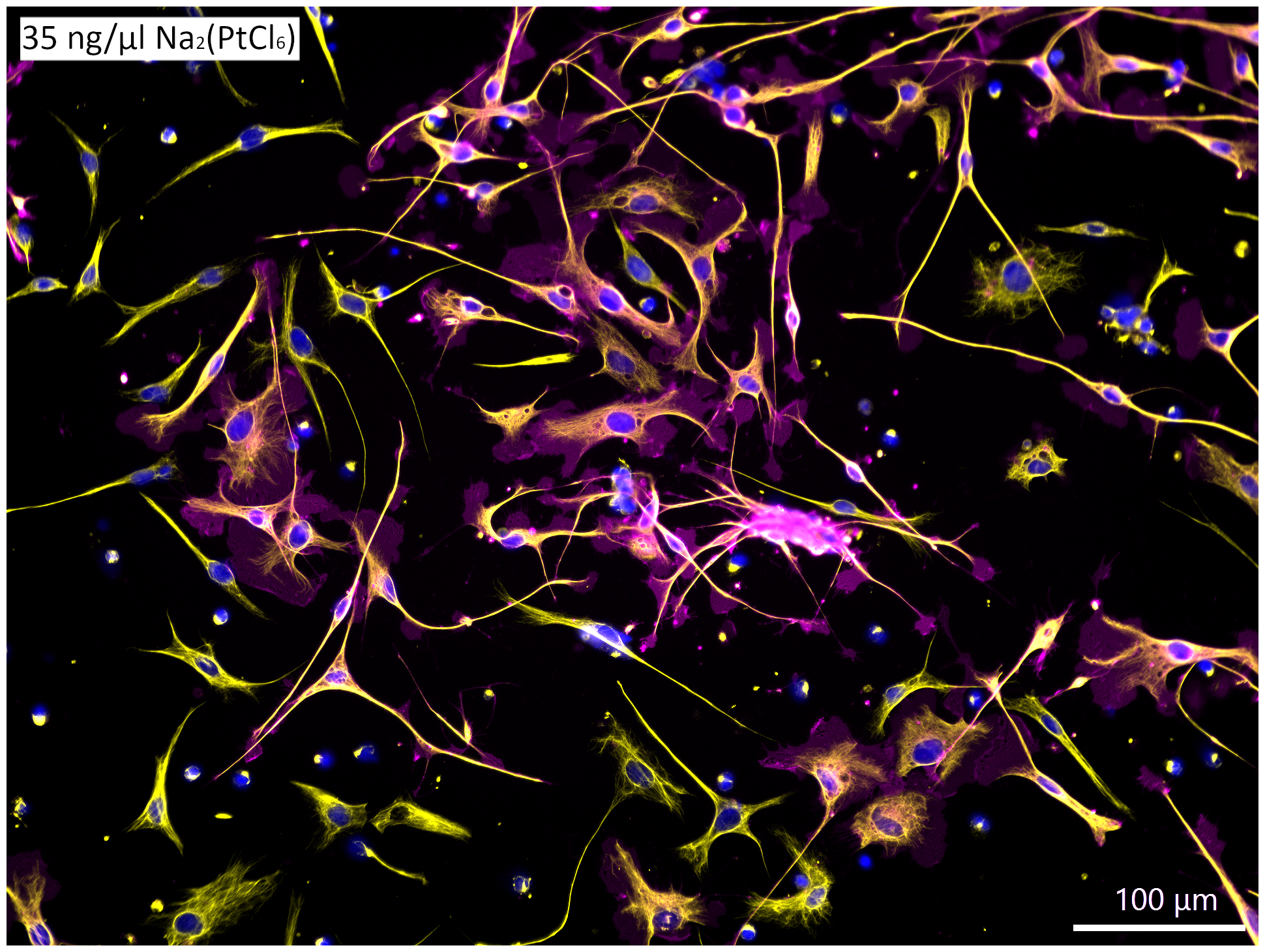

Supplement: S5 Fig — Representative SGC culture assay with 35 ng/μl Na2(PtCl6). (TIF) [file pone.0307973.s006.tif]

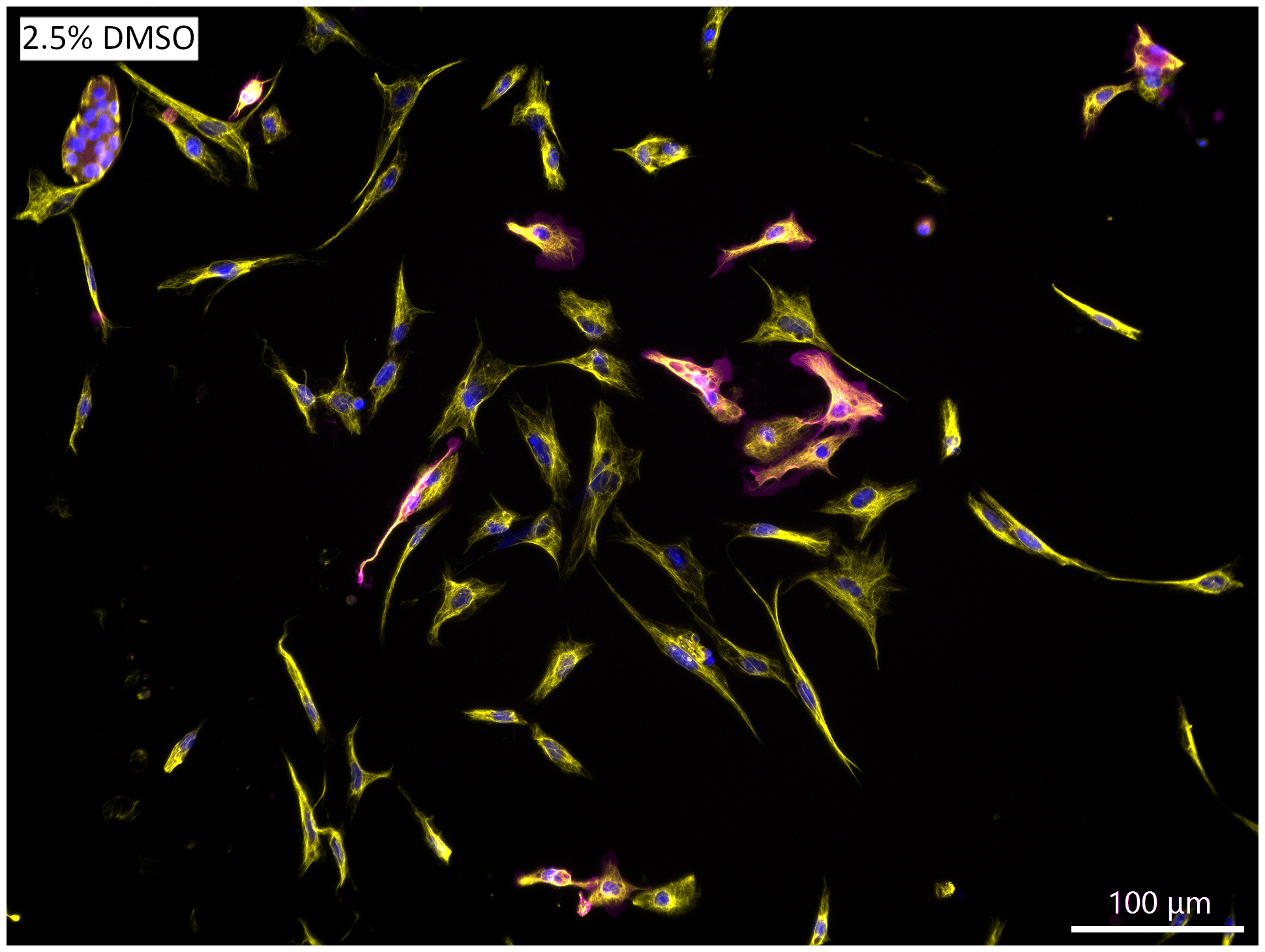

Supplement: S6 Fig — Representative SGC culture assay with 2.5% DMSO as cell death control. (TIF) [file pone.0307973.s007.tif]
